# Moving towards clinical trials for mitochondrial diseases

**DOI:** 10.1002/jimd.12281

**Published:** 2020-09-02

**Authors:** Robert D.S. Pitceathly, Nandaki Keshavan, Joyeeta Rahman, Shamima Rahman

**Affiliations:** ^1^ Department of Neuromuscular Diseases UCL Queen Square Institute of Neurology and The National Hospital for Neurology and Neurosurgery London UK; ^2^ Mitochondrial Research Group UCL Great Ormond Street Institute of Child Health London UK; ^3^ Metabolic Unit Great Ormond Street Hospital for Children NHS Foundation Trust London UK

**Keywords:** antioxidants, clinical trial, gene therapy, mitochondrial biogenesis, mitophagy, nucleosides, primary mitochondrial disease, treatment

## Abstract

Primary mitochondrial diseases represent some of the most common and severe inherited metabolic disorders, affecting ~1 in 4,300 live births. The clinical and molecular diversity typified by mitochondrial diseases has contributed to the lack of licensed disease‐modifying therapies available. Management for the majority of patients is primarily supportive. The failure of clinical trials in mitochondrial diseases partly relates to the inefficacy of the compounds studied. However, it is also likely to be a consequence of the significant challenges faced by clinicians and researchers when designing trials for these disorders, which have historically been hampered by a lack of natural history data, biomarkers and outcome measures to detect a treatment effect. Encouragingly, over the past decade there have been significant advances in therapy development for mitochondrial diseases, with many small molecules now transitioning from preclinical to early phase human interventional studies. In this review, we present the treatments and management strategies currently available to people with mitochondrial disease. We evaluate the challenges and potential solutions to trial design and highlight the emerging pharmacological and genetic strategies that are moving from the laboratory to clinical trials for this group of disorders.

## 1 INTRODUCTION

Primary mitochondrial diseases are recognised as some of the most common and severe inherited metabolic disorders, affecting ~1 in 4,300 live births.^1^ They are defined as genetic diseases in which mutations primarily or secondarily lead to dysfunction of oxidative phosphorylation (OXPHOS) or other disturbances of mitochondrial structure and function, including perturbed mitochondrial ultrastructure.^2^ Mitochondrial disorders are inherently heterogeneous at the genetic, mechanistic, and clinical level. Defects of nearly 400 genes across two genomes have been linked to primary mitochondrial diseases.^3^ Patients may exhibit manifestations in almost any organ and tissue in the body, leading to enormous diagnostic challenges.

The complexity of mitochondrial disease contributes to a lack of therapeutic options for patients. With the exception of a handful of vitamin and cofactor biosynthesis and transporter defects, nearly all mitochondrial disorders currently lack disease‐modifying curative therapies. Contributing factors to this current state include a poor understanding of disease pathomechanisms, inadequate disease models, the inaccessibility of the double‐membraned mitochondrion, and the logistical challenges of conducting clinical trials for ultra‐rare disorders.^2^ However, the current outlook for mitochondrial disease therapeutics is encouraging, with dozens of therapies in development and reaching clinical trials. Disease modelling has also improved significantly, and preclinical studies have been performed in mouse models of multiple mitochondrial DNA (mtDNA) deletions, a mt‐tRNA point mutation, mtDNA depletion syndrome (MDDS), epileptic encephalopathy, OXPHOS defects, coenzyme Q_10_ (CoQ_10_) biosynthesis defects, and mitochondrial translation defects.^4^, ^5^, ^6^, ^7^, ^8^, ^9^ Table 1 summarises a selection of the active mitochondrial disease clinical trials currently listed in clinicaltrials.gov.

**TABLE 1:** Ongoing^a^ clinical trials targeting primary mitochondrial disease

Therapy	Clinical trial identifier	Phase	Mechanism of action	Disorder	Age range (years)	Primary outcomes measure(s)	Status
EPI‐743	NCT01370447	II	Mitochondrial Redox Modulator	PMD	1+	Change in neuromuscular function, IAE, NPMDS	Active, not recruiting
Vatiquinone (PTC743, EPI‐743)	NCT04378075	II	Mitochondrial Redox Modulator	PMD with Refractory Epilepsy	≤18	Change from baseline in number of observable motor seizures per 28 days, number of disease‐related hospital days, number of participants with status epilepticus	Not yet recruiting
Vincerinone (EPI‐743)	NCT02352896	II	Mitochondrial Redox Modulator	Leigh Syndrome	1–18	Long term effect on disease severity measured by NPMDS	Active, not recruiting
KH176 (Sonlicromanol)	NCT04165239	II	Mitochondrial Redox Modulator	MELAS, MIDD, MM, PMD	18+	Cognitive function: attention domain	Recruiting
Idebenone (Raxone)	NCT02774005	IV	Mitochondrial Redox Modulator	LHON	12+	Proportion of eyes with clinically relevant recovery of visual acuity from baseline	Active, not recruiting
Idebenone (Raxone)	NCT02771379	PASS	Mitochondrial Redox Modulator	LHON	Child, Adult, Older Adult	Long‐term safety profile ‐ IAE	Recruiting
Nicotinamide Riboside	NCT03432871	N/A	NAD Modulator Mitochondrial Biogenesis Enhancer	MELAS, MM, PEO, PMD	18‐70	Bioavailability—pharmacokinetics. Safety—IAE, change in blood analytes, temperature, blood pressure, pulse. Mitochondrial biogenesis^31^P‐MRS, respiratory chain enzyme analysis, mtDNA copy number	Recruiting
KL1333	NCT03888716	I	NAD Modulator Mitochondrial Biogenesis Enhancer	MELAS, MM, MRCD, PMD, HV	18‐75	IAE, ECG, incidence of abnormal vital signs, incidence of abnormal physical examinations	Recruiting
REN001	NCT03862846	I	Mitochondrial Biogenesis Enhancer	MM	16+	IAE	Active, not recruiting
ABI‐009 (Nab‐sirolimus)	NCT03747328	II	Inhibition of Mitophagy	Leigh/ Leigh‐like Syndrome	2–17	IAE, GMFM	Not yet recruiting
L‐Citrulline	NCT03952234	I	Nitric Oxide Precursor	MELAS	18‐65	Maximal tolerable dose, IAE	Not yet recruiting
Sodium Phenylbutyrate	NCT03734263	I/II	Inhibition of Pyruvate Dehydrogenase Kinase	PDHC Deficiency	0.25‐18	Blood lactate levels	Recruiting
Dichloroacetate	NCT02616484	III	Inhibition of Pyruvate Dehydrogenase Kinase	PDHC Deficiency	0.5‐17	Observer Reported Outcome (ObsRO) measure of health, IAE	Recruiting
Thymidine and Deoxycytidine	NCT03639701	I/II	Nucleosides	Myopathic Thymidine Kinase 2 Deficiency	All	Liver transaminase levels, lymphocyte count, creatinine, ECG, incidence of diarrhoea	Enrolling by invitation
EE‐TP	NCT03866954	II	Erythrocyte Encapsulated ERT	MNGIE	12+	Safety—IAE, laboratory indices, vital signs. Pharmacodynamics—changes in plasma and urine thymidine and deoxyuridine levels. Efficacy— change in body mass index.	Not yet recruiting
CD34+ cells enriched with MNV‐BLD	NCT03384420	I/II	Biological	PMD, PS	Child, Adult, Older Adult	IAE, IPMDS QoL questionnaire	Enrolling by invitation
scAAV2‐P1ND4v2	NCT02161380	I	Gene Therapy	LHON	15+	IAE	Recruiting
GS010 (rAAV2/2‐ND4)	NCT02064569	I/II	Gene Therapy	LHON	18+	IAE	Active, not recruiting
GS010 (rAAV2/2‐ND4)	NCT03293524	III	Gene Therapy	LHON	15+	BCVA	Active, not recruiting
GS010 (rAAV2/2‐ND4)	NCT03406104	III	Gene Therapy	LHON	15+	Long term follow up of gene therapy—IAE	Recruiting
rAAV2‐ND4	NCT03153293	II/III	Gene Therapy	LHON	10‐65	BCVA, computerised visual field	Active, not recruiting

Abbreviations: BCVA, best corrected visual acuity; CPET, cardiopulmonary exercise testing; ECG, electrocardiogram; ERT, enzyme replacement therapy; GMFM, gross motor function measure; HV, healthy volunteers; IAE: incidence of adverse events; IPMDS, international paediatric mitochondrial disease scale; LHON, Leber hereditary optic neuropathy; MDDS, mitochondrial DNA depletion syndrome; MELAS, mitochondrial encephalopathy lactic acidosis and stroke‐like episodes; MIDD, maternally inherited diabetes and deafness; MM, mitochondrial myopathy; MNGIE, mitochondrial neurogastrointestinal encephalopathy; MRCD, mitochondrial respiratory chain deficiency; MRS, magnetic resonance spectroscopy; NPMDS, Newcastle paediatric mitochondrial disease scale; PASS, post‐authorisation safety study; PDHC, pyruvate dehydrogenase complex; PEO, progressive external ophthalmoplegia; PS, Pearson syndrome; QoL, quality of life.

a Selection of clinical trials for primary mitochondrial disease (PMD) listed in https://clinicaltrials.gov accessed May 22, 2020.

## 2 TREATABLE DISORDERS

Although the vast majority of mitochondrial diseases lack licensed therapies, a subset are responsive to treatment; specifically, single‐gene disorders of cofactor transport and metabolism. Vitamin and other organic cofactors, including thiamine, riboflavin, biotin and CoQ_10_, are required to catalyse the enzyme reactions necessary for OXPHOS and other mitochondrial pathways. In disorders of cofactor transport and metabolism, cofactor replacement at pharmacological doses has the potential to enhance downstream energy production. Deficiency of the SLC19A3 thiamine transporter is a relatively uncommon cause of Leigh syndrome, but prompt treatment with high dose biotin and thiamine is associated with an excellent clinical outcome.^10^ Riboflavin (vitamin B2) is a precursor of two flavocoenzymes flavin adenine dinucleotide (FAD) and flavin mononucleotide (FMN) which act as cofactors for more than 90 human enzymes, many of which are localised in the mitochondrion. Mitochondrial disorders that have been reported to respond to riboflavin supplementation include deficiencies of ACAD9, AIFM1 and complex I subunits located near the FMN binding site such as NDUFV1 and NDUFV2, as well as deficiency of FAD synthase, the only known disorder of riboflavin metabolism.^11^, ^12^ Furthermore, the riboflavin transporter disorders (Brown‐Vialetto‐Van Laere syndrome) may mimic mitochondrial disease, including deficiencies of respiratory chain enzymes.^13^, ^14^ Ten disorders of the biosynthesis of CoQ_10_, a non‐vitamin organic cofactor, are known and some cases may respond to supplementation with pharmacological doses of CoQ_10._ ^15^ Positive responses to treatment have been reported for defects of COQ2 and COQ8B (previously known as ADCK4).^16^, ^17^ However, there have been disappointing outcomes in other primary CoQ_10_ deficiencies, particularly those affecting the brain and with prenatal onset such as deficiencies of COQ4 and COQ9.^18^, ^19^ The lack of clinical response to CoQ_10_ supplementation in these patients has led to a search for new compounds to treat CoQ_10_ biosynthesis disorders. One promising strategy still in preclinical development is to use alternative benzoquinone ring precursors, including 4‐hydroxybenzoate and its analogues (such as 2,4‐dihydroxybenzoic acid), *p*‐coumarate, vanillic acid, resveratrol and kaempferol, to bypass the block in CoQ_10_ biosynthesis.^20^

## 3 SUPPORTIVE CARE

The current clinical management of mitochondrial disorders relies almost entirely on symptomatic treatment. Although vitamins and cofactors are frequently prescribed to patients with primary mitochondrial diseases, sometimes as a ‘cocktail’, there is no proven efficacy for these agents outside the disorders of vitamin/cofactor metabolism and transport described above.^21^ Symptomatic therapies are specific to the manifestations of the patient. For example, seizures may be treated with anti‐epileptic drugs or a ketogenic diet, cardiac dysfunction can be treated with drugs including β‐blockers, renal insufficiency may require haemodialysis, hearing aids or cochlear implants may be needed for patients with hearing loss, and brow suspension surgery may be indicated to improve vision or for cosmesis in patients with severe ptosis. Organ transplantation is also an option, but should be considered carefully in cases of multi‐system disease.^22^

## 4 BRIDGING THE TRANSLATIONAL GAP: THE LONG ROAD TO CLINICAL TRIALS

In 2012, a Cochrane Systematic Review of Treatments concluded that there was no clear evidence supporting the use of any intervention in mitochondrial disorders.^21^ Twelve trials fulfilled the entry criteria to the review, which included randomised controlled trials (including cross‐over studies). Since then, there have been significant advances in therapy development for mitochondrial diseases, as outlined below. However, there remains no licensed disease‐modifying therapies for patients. This might partly reflect a lack of efficacy of the interventions studied. However, other factors may have contributed to the failure of these trials. Trial design in mitochondrial diseases has historically been hampered by the lack of natural history data, given their relative rarity and heterogeneity, and validated biomarkers and outcome measures that correlate with disease progression. Progress is finally being made in these areas, as discussed in the following sections.

### 4.1 Natural history studies

Before attempting to design a clinical trial, it is important to possess a good understanding of the natural history of the disease. This includes appreciation of the age of onset of symptoms, the major clinical features as they evolve and any variation in disease severity in the patient cohort, together with morbidity and mortality outcomes.^23^ This has been undertaken on a large scale for several mitochondrial disorders to date.^24^, ^25^, ^26^, ^27^, ^28^, ^29^, ^30^ The therapeutic time window defines a duration of time within which a putative treatment may achieve its intended benefit, that is, duration between establishing the diagnosis and the time‐point after which disease severity is too great to achieve the desired outcome. It is important to determine whether there is any genotype‐phenotype correlation that modifies this therapeutic window. Since mitochondrial disorders often have a long delay to diagnosis^31^ many patients who are index cases within families will already be symptomatic by the time they are diagnosed. Neurological disease involvement is often severe and brain injury that accompanies metabolic decompensations such as stroke‐like episodes often results in progressive neurodisability.^32^ The utility of disease modifying treatments in patients with severe disease is therefore unclear. The situation for patients who are deemed to be at high risk of developing disease, for example siblings of affected patients who have been diagnosed pre‐symptomatically, should be more straightforward, but this is not always the case. For some mitochondrial disorders, individuals with identical genotypes may present with vastly differing clinical features^33^ and for patients with pathogenic variants in mtDNA, disease severity is dependent on the degree of mutation load in affected tissues.^34^

### 4.2 Selection of optimal outcome measures

Selection of appropriate outcome measures is one of the most important factors in enabling a clinical trial to determine the efficacy of a novel therapy correctly. Optimal outcome measures should be robust, sensitive, specific, validated for mitochondrial disease and clinically meaningful. Development of such measures has been particularly challenging given the clinical variability of mitochondrial diseases (even within genetically homogeneous groups), their relapsing‐remitting course and variable progression, which tends to occur over years to decades in adults, unlike most clinical trials that are conducted over much shorter time periods. Nevertheless, collaborative efforts are underway to overcome these hurdles, including the development of large national and international ‘trial‐ready’ patient cohorts,^35^, ^36^, ^37^ and consensus recommendations on quality of life and clinical outcome scales and potential mitochondrial biomarkers to monitor efficacy.^38^, ^39^, ^40^ Prospective natural history studies may help to identify appropriate biomarkers that could be used to assess the response to the trial drug. Presently however there is a lack of biomarkers that are sensitive enough to be used for this purpose in large patient cohorts.^41^, ^42^ Recently, there have been promising advances in the utility of multi‐omic approaches to identify novel biomarkers, however these are yet to be validated in patients.^42^

### 4.3 Clinical trial design

**FIGURE 1:**
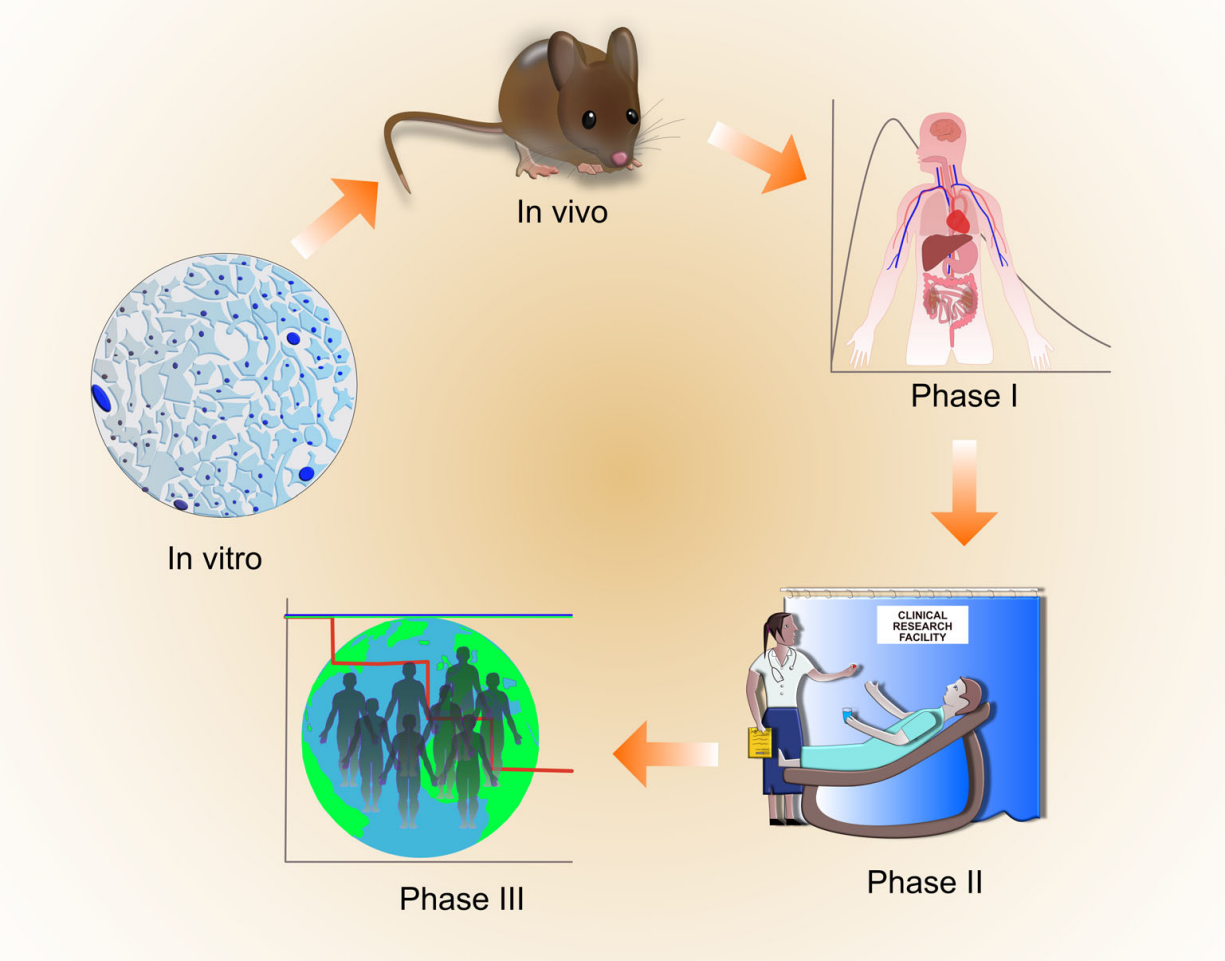
**Translational pipeline.** Candidate drugs are first investigated in vitro for example, in patient cell lines before in vivo toxicity and efficacy studies in appropriate animal models of disease are undertaken. Clinical trials include phase I studies, in which the candidate therapy is administered to patients or healthy volunteers to assess safety and tolerability, as well as drug pharmacokinetics. Phase II studies assess safety and efficacy of the drug in a small number of patients. Phase III studies assess safety and efficacy of the drug in a larger number of patients with defined outcome measures

It is essential that patients who are to be enrolled into clinical trials seeking to treat primary mitochondrial diseases possess a genetic diagnosis which confirms their eligibility, since there are many other diseases that are not considered primary mitochondrial diseases which may be associated with secondary mitochondrial dysfunction. Traditional clinical trials typically have three main phases with escalating numbers of patients in each phase. Phase I studies, performed in small numbers of individuals (often healthy volunteers), test safety, pharmacokinetics and dosing whilst phase II trials begin to address effectiveness, with further assessment of safety. Phase III trials usually involve hundreds (or thousands for common disorders) of patients at multiple centres, and compare effectiveness of the drug under investigation to the standard of care, together with further safety studies and evaluation of side effects (Figure 1). The ideal clinical trial should be adequately powered, statistically valid, randomised, double‐blinded, placebo controlled and include a large group of patients with the same genetic defect (similar mutation load if mtDNA), the same clinical presentation, the same biochemical findings and at a similar stage of disease progression. It is clearly impossible to achieve all of these aims in the context of mitochondrial disease, a collection of hundreds of ultra‐rare disorders with variable and unpredictable progression. However, this does not mean that good quality trials cannot be performed for this group of patients, and care should be taken to design trials that will answer the issue being addressed.^43^ One aspect of mitochondrial disease that has remained virtually unchanged is disease prognosis, which is still disappointing. More than 80% of disorders have an onset in childhood, and the central nervous system is the most frequently involved. Overall mortality is high, with close to 75% of mitochondrial disorders for which natural history data have been reported having a life expectancy of under 10‐years.^23^ This implies that for a majority of disorders recruitment of children under the age of 12 years to a clinical trial is likely to be limited and that in order to achieve adequate statistical power for studies involving this age group, multicentre collaboration will be necessary. Moreover, alternative trial designs need to be explored, owing to small numbers of patients affected by ultra‐rare disorders.^43^ Another important initiative is to identify potential barriers to participating in clinical trials for patients affected by primary mitochondrial diseases.^44^

## 5 PHARMACOLOGICAL STRATEGIES FOR MITOCHONDRIAL DISEASES

**FIGURE 2:**
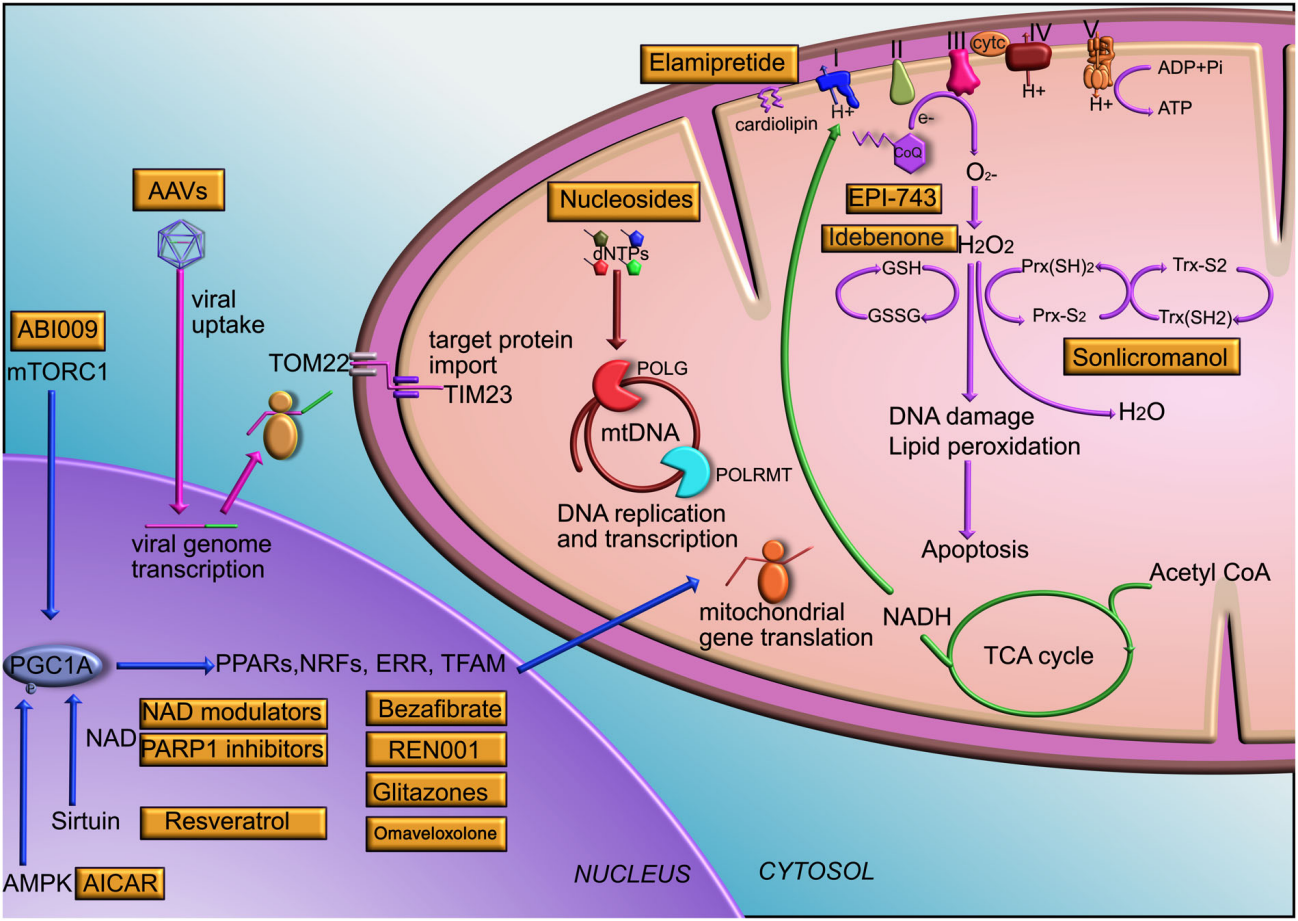
**Mechanisms of action of emerging therapies.** Drugs affecting mitochondrial biogenesis act on the PGC1α pathway. PGC1α is a master transcriptional coactivator of several transcription factors including PPARα,δ,γ, NRF1,2, ERR and TFAM. PGC1α is activated by phosphorylation by AMPK and deacetylation by NAD^+^‐dependent sirtuin, and is also controlled by mTOR. Drugs acting on these pathways include AICAR which activates AMPK, resveratrol which activates sirtuin, NAD^+^ modulators and PARP1 inhibitors which increase NAD^+^ levels, rapamycin and ABI009 which act on mTORC1, bezafibrate which activates PPARα, REN001 which activates PPARδ, glitazones which activate PPARγ and omaveloxolone which activates NRF2. Gene therapy vectors for example, AAVs transduce target cells by first being endocytosed at the plasma membrane. The viral genome is released in the nucleus where it forms an episome and is transcribed by target cell transcriptional machinery. mRNAs are translated in the cytosol. The nascent protein contains a mitochondrial targeting sequence which enables entry into mitochondria by interacting with the TOM22/TIM23 complex. Nucleoside based trial drugs are currently only applicable to one subtype of MDDS, namely thymidine kinase 2 deficiency. Several candidate therapies act on pathways related to the production of ROS, such as superoxide and hydrogen peroxide. Their intermediates have important cellular signalling functions, but also contribute to disease pathophysiology and cell death in mitochondrial disease. Levels of ROS are controlled by the glutathione and peroxidoredoxin/thioredoxin pathways. EPI743 and idebenone are both CoQ analogues which are thought to affect glutathione levels and Sonlicromanol acts on the peroxidoredoxin/thioredoxin pathway. Key: AAV, adeno‐associated virus; cytc, cytochrome *c*; CoQ, coenzyme Q; AMPK, AMP activated protein kinase; GSH, glutathione (reduced); GSSG, glutathione (oxidised); ERR, oestrogen related receptor; MDDS, mitochondrial DNA depletion syndrome; mRNA, messenger RNA; mTORC1, mechanistic target of rapamycin complex 1; NAD, nicotinamide adenine dinucleotide; NRF, nuclear respiratory factor; PARP1, poly(ADP‐ribose) polymerase 1; PGC1α, peroxisome proliferator‐activated receptor gamma coactivator 1‐alpha; PPAR, peroxisome proliferator‐activated receptor; POLG, polymerase gamma; Prx, peroxiredoxin; ROS, reactive oxygen species; TCA, tricarboxylic acid; TFAM, transcription factor A, mitochondrial; TIM, translocase of inner membrane; TOM, translocase of outer membrane; Trx, thioredoxin

**FIGURE 3:**
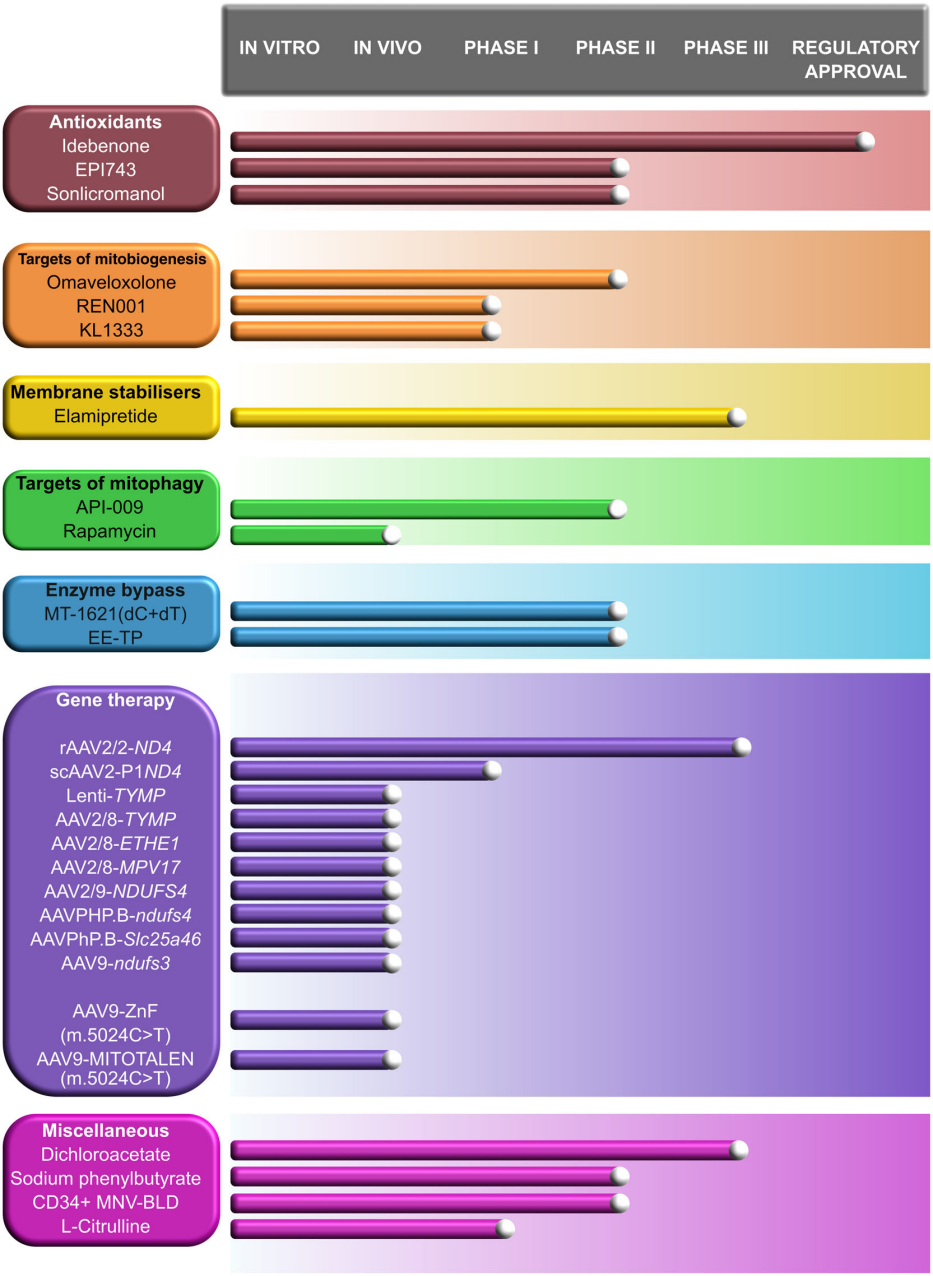
Progress in clinical trial development for mitochondrial disorders including trials that have been completed and those that are currently recruiting

A pharmacological therapy can be defined as a chemical compound which elicits a mechanistic change to a cellular component. In the context of mitochondrial diseases, these can include vitamin and cofactor administration discussed above, but also comprises a variety of novel compounds, many of which are in clinical trials. A summary of mechanisms of action of drugs discussed in the following sections is depicted in Figure 2. Figure 3 illustrates the clinical trials landscape of emerging therapies for mitochondrial diseases.

### 5.1 Antioxidant approaches

Under physiological conditions, reactive oxygen species (ROS) are important signalling molecules, with wide‐ranging effects on cell migration, viability, and differentiation.^2^ However, the pathological over‐production of ROS leads to a perturbed mitochondrial network, increased phospholipid peroxidation, activation of an inflammatory response, and hyper‐activation of apoptosis.^45^, ^46^ A number of antioxidant agents have been developed, with the aim of ameliorating the deleterious effects of ROS on membrane integrity, DNA damage, and activation of apoptotic pathways. A cautionary note when considering antioxidant therapy is that prolonged exposure to excessive antioxidant doses may potentially have deleterious consequences. One study reported worse survival of *cox15* knock‐out mice treated with N‐acetylcysteine.^47^

Idebenone was among the first artificial mitochondrial antioxidants to be developed and was reported to improve respiration in rat brain mitochondria and counteract lipid peroxidation and cerebral vascular lesions.^48^ Idebenone is a synthetic analogue of CoQ_10_ with a shorter chain, granting greater solubility for improved pharmacokinetics and an ability to cross the blood brain barrier, making it attractive for the treatment of mitochondrial diseases affecting the central nervous system. In clinical trials, idebenone demonstrated mild improvement of neurological phenotype in Friedreich ataxia and was found to mildly improve visual acuity and colour contrast sensitivity in Leber hereditary optic neuropathy (LHON).^49^ It is currently approved for treating LHON in Europe but is not FDA approved. A clinical trial of idebenone in MELAS (NCT00887562) has been completed, with a primary outcome measure of mean change in cerebral lactate concentration measured by magnetic resonance spectroscopy (MRS), but the results have not been published. Another CoQ_10_ analogue EPI‐743, claimed to have 1000 to 10 000‐fold greater potency than both CoQ_10_ and idebenone, has been investigated in open‐label trials in Leigh syndrome, with inconclusive results.^50^, ^51^ These CoQ_10_ analogues appear to function by restoring redox balance and counteracting ROS, notably by increasing glutathione production.^50^, ^51^

Sonlicromanol (previously known as KH176) is another ROS‐redox modulator which has been shown to decrease cellular ROS levels and protect fibroblast cell lines, derived from patients harbouring mutations in nuclear encoded complex I subunits, against redox perturbation by targeting the thioredoxin/peroxiredoxin system.^52^ Long‐term Sonlicromanol treatment of *Ndufs4* ^*−/−*^ mice, a mammalian model for Leigh syndrome, retained brain microstructural coherence in the external capsule and normalised lipid peroxidation in this area and the cerebral cortex.^53^ It also significantly improved rotarod and gait performance and decreased retinal ganglion cell degeneration in the mice. A recent double‐blind, randomised, placebo‐controlled, two‐way crossover phase IIa study (the KHENERGY study) of Sonlicromanol in 18 adults with the m.3243A > G *MT‐TL1* mutation showed that the drug was well‐tolerated with no treatment‐emergent adverse events.^54^ Although no significant improvement in gait parameters or other outcome measures was obtained, a positive effect on alertness and mood was detected. A phase IIb study of Sonlicromanol in adult m.3243A > G disease is currently recruiting (KHENERGYZE, NCT04165239).

### 5.2 Harnessing mitochondrial biogenesis

Mitochondrial biogenesis refers to an increase in mitochondrial mass. It is activated by a large number of physiological stimuli including fasting, cold exposure and exercise, with the ultimate aim of matching energy supply and demand. Pathways related to mitochondrial biogenesis centre on the Peroxisome proliferator‐activated receptor gamma coactivator 1‐alpha (PGC1α), a transcriptional coactivator that interacts and boosts activity of several metabolism‐related transcription factors.^55^ PGC1α is a target for Sirtuin1 (Sirt1), a nicotinamide adenine dinucleotide (NAD^+^)‐dependent protein deacetylase. Irrespective of the underlying molecular mechanism, the downstream consequence of impaired OXPHOS is reduced synthesis of ATP. Thus, pharmacological stimulation of mitochondrial biogenesis via manipulation of the PGC1α axis represents a potential therapeutic strategy in primary mitochondrial diseases, and several small molecules have been identified that exert their effect through this pathway. Examples include bezafibrate,^56^ glitazones,^57^ resveratrol,^58^ omaveloxolone,^59^ aminoimidazole‐4‐carboxamide ribonucleotide (AICAR),^60^ NAD^+^ modulators (eg, tryptophan, nicotinic acid, nicotinamide, nicotinamide riboside, KL1333), poly(ADP‐ribose) polymerase 1 (PARP1) inhibitors,^61^, ^62^ REN001, and decanoic acid.^63^ Although there is promise from animal and patient‐derived models for some of these compounds,^61^, ^62^, ^63^ others have generated data that have not been reliably reproducible or suggested toxicity in mice.^60^, ^64^, ^65^

Three human clinical trials investigating agents purported to increase mitochondrial biogenesis have been completed recently. An open‐label observational study of bezafibrate in six patients with mitochondrial myopathy caused by the m.3243A>G mutation showed improvement in cytochrome *c* oxidase (COX)‐deficient muscle fibres and cardiac function, with no clinically significant adverse events.^66^ Although liver function was unaffected, fibroblast growth factor 21 (FGF21) and growth and differentiation factor 15 (GDF15) levels increased and fatty acid and amino acid metabolism was altered. The authors concluded that although bezafibrate might have short‐term benefits, concerns about the longer‐term consequences of metabolic reprogramming potentially restrict its application in mitochondrial diseases. A double‐blind, randomised, placebo‐controlled, cross‐over study of resveratrol supplementation in patients with mitochondrial myopathies and skeletal muscle fatty acid oxidation disorders (NCT03728777) has been completed recently but the results have not been reported yet.

A phase II randomised double‐blinded placebo controlled clinical trial was recently completed to assess safety and efficacy of omaveloxolone in 53 patients with mitochondrial myopathy (NCT02255422). Omaveloxolone induces mitochondrial biogenesis by activating NRF2. Patients on the treatment arm received the drug in six dose escalations. The primary and secondary outcome measures included peak cycling exercise workload and distance travelled in the six‐minute walk test (6MWT). Results indicated that omaveloxolone did not meet its primary and secondary endpoints although it was well tolerated in the majority of subjects.^67^

An open label phase Ib study evaluating the safety and tolerability of REN001 (NCT03862846), a peroxisome proliferator‐activated receptor delta (PPARδ) agonist, in patients with primary mitochondrial myopathy, is currently underway, while a phase Ia/Ib trial of KL1333 (NCT03888716), a modulator of cellular NAD^+^ levels, is being conducted in people with primary mitochondrial diseases.

Another group of therapies currently undergoing clinical trials involved the restoration of redox balance in mitochondria as a means of stimulating mitochondrial biogenesis. Disrupted redox in the form of the NADH/NAD^+^ ratio has wide‐ranging cellular effects, including epigenetic changes via the action of NAD^+^‐dependent sirtuins and intramitochondrial changes in ROS production and calcium signalling. Supplementation with the vitamin B3 derivative nicotinamide riboside, an NAD^+^ precursor, stimulated mitochondrial biogenesis and improved mitochondrial disease phenotypes in two mouse models.^61^, ^62^ An open label study of niacin (nicotinic acid) has recently been conducted in patients with mitochondrial myopathy, with post‐treatment increased NAD^+^ levels detected in blood and muscle tissue, alongside enhanced mitochondrial biogenesis.^68^ Finally, an open label study of nicotinamide riboside in mitochondrial biogenesis is currently recruiting patients (NCT03432871).

### 5.3 Stabilisation of cardiolipin

Elamipretide (also known as SS‐31, MTP‐131, and Bendavia) is a mitochondrially‐targeted Szeto‐Schiller tetrapeptide that is reported to decrease the production of ROS. Although the mechanism of action is not entirely clear, Elamipretide is thought to exert its effects by stabilising cardiolipin and thus improving the efficiency of the mitochondrial respiratory chain. Elamipretide recently completed a phase III clinical trial (MMPOWER‐3, NCT03323749), following a phase II study that demonstrated a clinically meaningful but not statistically significant increase in exercise performance (6MWT) after 4 weeks of daily subcutaneous treatment in patients with primary mitochondrial myopathy.^69^ Safety results showed that treatment with Elamipretide was well tolerated, with most adverse events being mild to moderate in severity. Unfortunately, a press release from the manufacturer in December 2019 confirmed that the phase III study did not meet its primary endpoints assessing changes in the 6MWT and Primary Mitochondrial Myopathy Symptom Assessment (PMMSA) Total Fatigue Score.^70^ Elamipretide has also recently completed a phase II randomised controlled blinded crossover study with open‐label follow‐up in 12 patients with Barth syndrome (TAZPOWER). The trial failed to meet its primary endpoint of improving 6MWT and patient‐reported fatigue scores during the first 12 weeks of the study which were blinded. However, there were statistically significant improvements in both outcomes at 36 weeks' follow up in the open‐label extension. In addition, during open‐label follow‐up echocardiographic assessments indicated an improvement in cardiac function as determined by increased mean stroke volume and left ventricular end‐diastolic volume.^71^

### 5.4 Targeting mitophagy

Mitophagy is the physiological maintenance of normal mitochondrial function by selective elimination of damaged mitochondria.^72^ Mitophagy is regulated by numerous cellular pathways, one of which includes mTOR (mechanistic inhibition of rapamycin).^73^ Rapamycin inhibits mTOR and was shown to prolong lifespan of the *Ndufs4* ^*−/−*^ Leigh syndrome mouse model.^74^ It has subsequently been shown to benefit a large number of cell‐based and in vivo mitochondrial disease models,^75^, ^76^, ^77^, ^78^, ^79^, ^80^ although the underlying mechanism is unclear. However, there was no evidence of benefit from rapamycin in a mouse model of CoQ_10_ deficiency,^81^ suggesting that it may not be a universal mitochondrial panacea. Observational studies in humans have yielded conflicting results. Four renal transplant patients with m.3243A>G disease were reported to have clinical improvement after their immunosuppression was changed from calcineurin inhibitors to rapamycin or everolimus^82^ whilst one child with Leigh syndrome apparently improved and another with MELAS deteriorated after commencing everolimus therapy.^83^ Formal clinical trials are needed, and an open‐label phase IIa Study to Evaluate the Safety, Tolerability, and Clinical Activity of ABI‐009 (the rapamycin derivative, Nanoparticle albumin‐bound Sirolimus) in patients with genetically confirmed Leigh or Leigh‐like Syndrome is currently ongoing but not yet recruiting (NCT03747328).

### 5.5 Enzyme bypass studies

Single‐peptide enzymes present in yeast and lower eukaryotes have been harnessed to bypass specific mitochondrial respiratory chain enzyme complexes. Examples include NADH reductase (Ndi1) and alternative oxidase (AOX) which have been expressed in cellular and Drosophila models to bypass complex I and complex III and IV defects, respectively.^84^, ^85^, ^86^, ^87^ AOX has also been expressed in two mouse models of mitochondrial disease, with apparently beneficial effects in *Bcs1l* ^*p.S78G*^ knock‐in mice but exacerbation of mitochondrial myopathy secondary to aberrant redox signalling in the skeletal muscle‐specific *Cox15* knockout mouse crossed with an AOX‐transgenic mouse.^47^, ^88^

More recently, an engineered enzyme LOXCAT, fusing a bacterial lactate oxidase (LOX) and catalase (CAT), was developed with the aim of decreasing reductive stress by oxidising extracellular lactate to pyruvate.^89^ Applying LOXCAT to cell lines with chemically‐induced and disease‐relevant genetic knockout models of mitochondrial respiratory chain dysfunction, in addition to human mutant fibroblasts, reduced extracellular lactate:pyruvate, normalised intracellular NADH:NAD^+^, increased ATP production by glycolysis and improved cell proliferation. Similar findings were observed in wild‐type and metformin‐treated mice following LOXCAT tail vein injections.

### 5.6 Hypoxia

Von Hippel‐Lindau (VHL) factor was identified as an effective suppressor of mitochondrial dysfunction through a CRISPR/Cas9 genome‐wide screen to search for repressors of antimycin‐induced complex III deficiency.^90^ The VHL ubiquitin ligase appears to exert its effects via activation of the hypoxic response pathway, negatively regulating hypoxia‐induced transcription factors (HIFs) during the hypoxic response. In‐vivo studies demonstrated an increased lifespan of *Ndufs4* ^*−/−*^ mice when exposed to chronic normobaric hypoxic (11% O_2_) conditions. Normoxic conditions reversed these beneficial effects, while hyperoxia was detrimental.^90^ Although these data are all preclinical, a speculative translational implication of these studies is that hyperoxia should be avoided in the mitochondrial patient in the ICU.^91^, ^92^

### 5.7 Nucleoside supplementation

Since mitochondrial dNTP pool imbalances contribute to the pathogenesis of MDDS, nucleoside precursor supplementation has been proposed as a method to correct these dNTP pool imbalances. In mice, substrate enhancement therapy by dT and dC administration in *Tk2* ^*−/−*^ mice improved mtDNA copy number and life span in the encephalomyopathic animals.^93^ Subsequently, 38 patients with TK2 deficiency were treated at multiple centres with oral pyrimidine nucleosides dC and dT on a compassionate‐use basis (NCT03701568). A retrospective analysis of these patients compared their outcomes to 68 published untreated cases and revealed a significant improvement in survival of treated patients compared to natural history data of untreated patients.^94^ No deaths were reported in treated patients. Ninety‐four percent of patients who received treatment were found to either have improved (especially in gross motor assessments) or remained stable (especially in respiratory and feeding assessments) regardless of the age of disease onset. The most common adverse effect was diarrhoea, followed by derangements in liver function and urolithiasis. A prospective open‐label phase II clinical trial of MT‐1621, a GMP grade combination of dC and dT, is now underway (NCT03845712). The primary aim is to assess safety of the drug treatment, with a secondary aim to assess efficacy including motor function, respiratory status and effects on growth and nutrition.

Nucleoside therapy has been proposed for other forms of MDDS. However, a major concern is that supplementation may potentially induce further dNTP imbalances causing unexpected and unpredictable side effects. Furthermore, there is evidence that mitochondria play a role in the toxicity of other nucleoside‐based therapies. For example, anti‐retroviral reverse transcriptase inhibitors such as 3′‐azido‐2′,3′‐dideoxythymidine (AZT) elicit deleterious effects on TK2 and DGUOK, leading to mitochondrial disease‐like side effects in patients.^95^ AZT and other nucleoside analogues used for the treatment of acquired immunodeficiency syndrome (AIDS) have also been found to be preferentially incorporated by POLG, resulting in gross mitochondrial genome instability.^96^ In fact POLG is the most sensitive DNA polymerase to nucleoside analogue inhibition after the retroviral reverse transcriptase.^97^ As a result, a secondary mitochondrial myopathy is an observed side effect of using these drugs in patients with AIDS.^98^ Thus, further studies of the effects of nucleoside therapy need to be conducted in different disease models of MDDS before considering clinical applicability.

### 5.8 Enzyme replacement therapy: Erythrocyte encapsulated thymidine phosphorylase

Mitochondrial Neurogastrointestinal Encephalopathy (MNGIE) is caused by bi‐allelic *TYMP* mutations resulting in a harmful accumulation of thymidine. In this disorder, enzyme replacement in the form of allogeneic haematopoietic stem cell transplantation (HSCT) has been shown to be effective in restoring thymidine phosphorylase activity and reducing thymidine levels to normal circulating levels in patients. However, a study of 24 patients who received HSCT demonstrated poor survival outcomes with deaths reported in >60% of cases.^99^ It is clear that a safer treatment is needed.

An alternative approach that is being investigated is delivery of enzyme replacement via erythrocyte encapsulation. In this strategy, patient erythrocytes are removed from circulation and treated ex vivo with recombinant thymidine phosphorylase using a red cell loader device to enable encapsulation of the enzyme within the erythrocytes. These cells are then infused back into the patient. A phase II open‐label trial in adults (NCT036866954) aims to investigate safety and efficacy of multi‐dose erythrocyte encapsulated thymidine phosphorylase (EE‐TP), utilising three dose levels to determine the minimum dose required to achieve metabolic correction on an individual patient basis.^100^ The primary endpoint for this study is the mean change in baseline body mass index after 2 years of treatment. Secondary endpoints include evaluation of adverse events, laboratory blood tests, ECGs, clinical observations, and physical examination.

## 6 GENETIC STRATEGIES FOR MITOCHONDRIAL DISEASES

In addition to novel small molecule therapies for mitochondrial disorders, a number of genetic therapies are also in development. Gene therapy seeks to correct the underlying defect of a genetic disease by delivering a normal copy of the mutated gene to affected individuals, and represents a potential cure, as it would rescue the molecular defect. The case for applying in vivo gene therapy to mitochondrial disorders is compelling. Solid organ transplantation for example, in liver (DGUOK deficiency, TYMP deficiency),^101^, ^102^ kidney (COQ2 deficiency)^103^ and heart (Kearns‐Sayre syndrome)^104^ has been used with variable success, but crucially does not correct disease in other affected organs such as the brain.^22^ Gene therapy has the potential to target multiple organs simultaneously, including the central nervous system, making it an attractive treatment strategy.

Different gene therapy approaches are being investigated for mitochondrial disorders, and can be grouped broadly into non‐viral and viral‐based methods. Non‐viral approaches include physical methods of cell membrane bombardment (hydrodynamic injection of DNA, biolistic methods),^105^, ^106^ chemical methods (micelles of cationic surfactants, rhodamine nanoparticles, liposomes),^107^, ^108^ and harnessing endogenous import machinery by means of mitochondrial targeting signal peptide (MTS)‐mediated translocation via the TOM22/TIM23 complexes.^109^ Many of the non‐viral methods are at preliminary in vitro stages and are limited by poor transfection efficiency, weak specificity to mitochondria or failure due to cytotoxicity.^110^

The most promising gene therapy vectors presently are adeno‐associated viral vectors (AAV) which belong to the parvovirus family but do not cause disease in humans. Naturally occurring AAVs occur in several subtypes each reflecting a unique pattern of tissue tropism (targeting).^111^ Recombinant AAV9 vectors represent an improvement on previous AAV vectors because they cross the blood brain barrier to transduce neurons well and provide a sustained effect after a single neonatal intravenous administration.^112^ They also retain the ability to target visceral organs, have a low risk of insertion into the host genome, low immunogenicity and low toxicity.^113^, ^114^, ^115^ AAV9 has already been applied to rodent models of various neurological and metabolic disorders including Hunter syndrome, Sanfilippo disease, Gaucher disease, GM1 gangliosidosis, Pompe disease and spinal muscular atrophy (SMA) with excellent efficacy.^116^, ^117^, ^118^, ^119^, ^120^, ^121^ Currently several AAV gene therapies are in clinical trials for a range of neurometabolic disorders including LHON (NCT02161380), Hunter syndrome (NCT03041324), GM1 gangliosidosis (NCT03952637), Pompe disease (NCT04174105), CLN2 disease (NCT00151216), Hurler syndrome (NCT02702115), AADC deficiency (NCT02852213) and Sanfilippo disease (NCT03612869). Currently, two gene therapies hold FDA approval, for the treatment of spinal muscular atrophy and retinal dystrophy.^122^, ^123^

### 6.1 Gene therapy for nuclear genes: Preclinical studies

AAV‐mediated gene therapy in mitochondrial disease has been tested mostly in mouse models, and overall, the results in mice have been promising.^124^, ^125^, ^126^, ^127^, ^128^ Gene therapy has been applied to a mouse model of MNGIE. Pyrimidine metabolism in mice is different to humans. Thus, in order to model disease in mice, a double knockout of both enzymes thymidine phosphorylase and uridine phosphorylase is required. These knockout animals (*Tymp* ^−/−^/*Upp1* ^−/−^) recapitulate the elevation of thymidine and deoxyuridine seen in MNGIE although they do not demonstrate liver or gastrointestinal disease. Both lentiviral and AAV‐based approaches have been trialled. For the lentiviral approach, haematopoetic progenitors were treated ex vivo with a lentivirus containing the *hTYMP* cDNA driven by the human phosphoglycerokinase promoter. A transduction efficiency of up to 28% was demonstrated using flow cytometry. Cells were then infused into partially myeloablated *Tymp* ^*−/−*^ */Upp1* ^*−/−*^ animals. After a period of 4‐weeks, there was a supranormal level of thymidine phosphorylase activity and normalisation of thymidine and deoxyuridine levels in peripheral blood as compared to sham treated animals.^129^ An alternative AAV‐based approach has been used to reconstitute thymidine phosphorylase activity by targeting mouse hepatocytes in vivo.^130^ In this study, an AAV2/8 recombinant vector was produced containing the *hTYMP* cDNA sequence driven by the liver specific thyroxine‐binding globulin promoter (TBG). Adult *Tymp* ^*−/−*^ */Upp1* ^*−/−*^ mice were treated with single intravenous injections of the AAV at doses ranging from 2 × 10^11^ to 2 × 10^12^ vector genomes (vg)/kg. Long‐term follow up of animals receiving the highest dose over 22 months revealed a reduction in the circulating thymidine and deoxyuridine levels which was sustained to the end of the period of follow‐up. There was also a resolution of liver intramitochondrial deoxyribonucleoside imbalances (elevated dTTP and low dCTP) that are seen in the knock‐out animals. However, in all dosage groups in long term follow‐up there was a reduction in transgene copy number and a consequent reduction in thymidine phosphorylase, but notably at the highest dose, enzyme activity was still supranormal. Overall, these studies demonstrate effective hepatic transduction with consequent clearance of thymidine.

An AAV2/8 recombinant vector has been used to ameliorate the ethylmalonic encephalopathy phenotype of *Ethe1* knock‐out mice.^126^ This knock‐out model recapitulated the biochemical and clinical features of ethylmalonic encephalopathy including reduced sulphur dioxygenase (SDO) activity in liver, COX deficiency in skeletal muscle and brain and reduced survival to 4 weeks' age. The transgene cassette used was AAV2/8‐TBG‐h*ETHE1* and route of administration was intracardiac. High doses of AAV vector (4 × 10^13^ vg/kg) were able to restore SDO activity, which correlated with lower levels of plasma thiosulphate levels, lower ethylmalonic acid levels and longer survival. Prolonged survival correlated with a higher transgene copy number in liver. COX activities also improved in skeletal muscle and brain after AAV treatment. Treated animals showed improved weight gain, normal motor activity and improved survival to beyond 6 months.

The *Mpv17* ^*−/−*^ mouse model of MDDS does not demonstrate liver disease at baseline despite the presence of liver mtDNA depletion, unless animals are maintained on a high‐fat (ketogenic) diet which induces weight loss, liver cirrhosis and hepatic failure. AAV2/8‐based gene therapy using the *hMPV17* cDNA sequence under control of the TBG promoter was used to treat *Mpv17* ^*−/−*^ mice.^124^ Doses of 4 × 10^12^ and 4 × 10^13^ vg/kg were administered by intravenous (retro‐orbital) delivery to 2‐month old knock‐out animals which were then maintained on a ketogenic diet for a further 2 months. Liver transaminase levels normalised in peripheral blood in the treated animals, and mtDNA copy number and liver OXPHOS activity were restored to wild‐type levels. Weight of the animals improved and liver cirrhosis was prevented, based on histological analysis.

AAV‐based gene therapy strategies have been trialled in a *Ndufs4* ^*−/−*^ mouse model of Complex I deficient Leigh syndrome. This mouse model demonstrated encephalopathy from P40, together with weight loss, abnormal gait and lethargy, with death at P50.^126^ Initially, AAV2/9‐CMV‐*hNDUFS4* was administered intravenously to neonatal mice and presymptomatic mice (P21) at a dose of 1 to 2 × 10^12^vg/mouse. Correction of complex I activity was best seen in the heart and skeletal muscle, with poor brain transduction and consequent persistence of complex I deficiency in the brain. Intravenous delivery was therefore unable to alter the survival of knock‐out animals. Intracerebroventricular administration of AAV2/9‐CMV‐*hNDUFS4* did not improve survival despite improving brain transduction. A combined intravenous/ intracerebroventricular approach achieved better overall brain and visceral organ transduction. Complex I dysfunction was rescued completely in skeletal muscle and heart but only partially in brain. Consequently, there was a modest improvement in survival of the animals to just over 80 days. Further analysis demonstrated that transduction in brain was limited to glial cells, not neurons, and was poor in the basal ganglia.

An alternative approach has been used to treat *Ndufs4* ^*−/−*^ mice utilising the PHP.B capsid to improve brain transduction via intravenous delivery.^131^ In biodistribution studies, AAV‐PHP.B was shown to transduce both neurons and glia effectively throughout the brain of *Ndufs4* ^*−/−*^ mice after single intravenous administration, in addition to transducing the visceral organs. AAV‐PHP.B containing the CBA‐*Ndufs4* cassette was injected into 1 month old knock‐out animals at a dose of 10^12^ vg/mouse. This improved the lifespan of knock‐outs from 0% survival at 75 days to 50% survival at 250 days' follow up. From the perspective of neurological function, improvements in paw clasping, grip strength, locomotor activity, and a significant reduction in seizures were observed in treated animals. In addition, restoration of Ndufs4 protein expression in the brain and visceral organs, complex I activity and supercomplex formation was demonstrated. Histologically, there was a reduction in neuroinflammatory markers and gliosis in the cerebellum, olfactory bulb and vestibular nuclei. This work demonstrated that systemically administered gene therapy might be an effective method of treating mitochondrial diseases that involve both brain and visceral organs. Furthermore, adequate multisystemic targeting is dependent on selecting a combination of a ubiquitously active promoter and an appropriate capsid that enables efficient viral uptake across different tissues.

AAV‐PHP.B gene therapy was also able to ameliorate the disease phenotype of a mouse model of a mitochondrial dynamics defect in Slc25a46 deficiency.^128^ Clinically, this defect causes hereditary sensory and motor neuropathy. Mice with the defect demonstrate ataxia, feeding difficulty and premature death. Intravenous neonatal administration of AAV‐PHP.B CMV‐*Slc25a46*‐*eGFP* administered at doses of 1 × 10^11^ and 2 × 10^11^ vg/g was able to transduce affected tissues including the cortex, cerebellum, sciatic nerve, restore Slc25a46 protein in affected tissues, reduce neuroinflammation and neuronal loss in the cerebellum and optic nerve. Furthermore, gene therapy improved the body weight, coordination and lifespan of the animals in a dose‐dependent manner. This approach needs further refinement before clinical translation since it has been shown that AAV‐PHP.B has a more restricted intravenous biodistribution in non‐human primates than it does in mice.^132^

AAV9 gene therapy has been applied recently to a skeletal muscle specific conditional *Ndufs3* knock‐out mouse model.^133^ Untreated knock‐out animals develop weight loss from 3 months and die prematurely. They also exhibit myopathic features including exercise intolerance and lactic acidosis, reduced complex I activity and compensatory increases in complex II and IV activities in skeletal muscle. AAV9 vectors containing the mouse *Ndufs3* cDNA driven by the CMV promoter were administered to *Ndufs3* ^*−/−*^ mice by retro‐orbital injection at a dose of 1.25 to 1.66 × 10^15^ vg/kg at a pre‐symptomatic age of 15 to 18 days as well as to symptomatic mice aged 2 months. In both cases, the mice showed improvements in body weight, motor coordination, muscle strength and increased survival. Laboratory investigations showed a restoration of Ndufs3 protein expression which persisted at 15 months of follow‐up, with normal complex I activity and skeletal muscle histochemical appearances. These data suggest that AAV9‐based gene therapy can reverse the pathophysiological changes in post‐symptomatic animals with myopathy due to complex I deficiency.

Gene therapy using viral vectors holds much promise based on preclinical data, but with the exception of LHON (see below), we are yet to see clinical translation in other mitochondrial disorders. Some of the challenges associated with clinical translation include ensuring sustained gene expression (ie, avoidance of transgene silencing) in target tissues and restrictions as to which patients could benefit from AAV treatments based on the presence of neutralising antibodies to AAV capsids in individual patients.^134^ In addition AAVs have a limited packaging capacity of up to ~4.7Kb thereby limiting its use as a strategy for disorders where the transgene to be delivered is too large.^135^ Gene therapy for rare diseases is becoming a form of personalised medicine for which the number of patients who could benefit from a specific therapy is likely to be small, and therefore the cost of the therapy, once commercialised, is likely to be high.^136^ Nevertheless it is encouraging to see that some of the more recent preclinical studies have been able to demonstrate effective reversal of disease pathology following post‐symptomatic gene transfer.

### 6.2 Curing the mitochondrial genome 1: Selectively destroying mutant mtDNA using zinc finger nucleases and mitoTALENS

Genome editing in the mitochondrial genome is more difficult, owing to problems accessing the double membraned‐mitochondrion and importing nucleic acids, which makes CRISPR‐Cas9 gene editing impossible using currently available methods. Another element of complexity for mitochondrial disorders caused by mtDNA mutations is the high copy number of the mtDNA molecule, with hundreds or even thousands of copies per cell, depending on the cell type. An individual patient's tissue may possess both normal mtDNA molecules as well as mtDNA molecules containing deleterious mutations, a situation known as heteroplasmy. This gives rise to the concept of mtDNA heteroplasmy thresholds where a critical proportion of mutated mtDNA molecules may need to be present before the overall mitochondrial function of a tissue is deficient.

Despite these challenges, genetic strategies for therapeutic mtDNA manipulation are being developed. The existence of heteroplasmy introduces a potential opportunity for treatment by selectively destroying mtDNA molecules which possess the mutation, using nucleases, thereby shifting heteroplasmy in favour of wild‐type mtDNA.^137^ Currently the most promising strategies to achieve shifts in heteroplasmy utilise zinc finger nucleases and mitochondrial transcription activator‐like effector nucleases (mitoTALENS). In proof‐of‐principle experiments, zinc finger nucleases have been engineered that selectively destroy mtDNA molecules harbouring specific mtDNA mutations.^138^, ^139^ Subsequently, combined approaches for delivery of zinc finger nucleases to cells using AAV9 vectors have been undertaken in a mouse mitochondrial cardiomyopathy model caused by a m.5024C>T mutation in the mitochondrial tRNA for alanine (mt‐tRNA^Ala^). These studies demonstrated a dose‐dependent improvement in mt‐tRNA^Ala^ expression in the mouse heart.^140^ Another nuclease‐based approach is the use of mitoTALENs which can be engineered to recognise specific DNA sequences in order to induce double‐stranded breaks for DNA degradation. MitoTALENS delivered to in vitro cellular models of mitochondrial disease selectively eliminated the common ~5 kb mtDNA deletion, thus shifting heteroplasmy in these patient‐derived cybrid lines.^141^ More recently, an AAV9‐based approach has been used to deliver a mitoTALEN specific to the m.5024C>T mutation driven by a CMV promoter to treat the mt‐tRNA^Ala^ cardiomyopathy mouse model. This approach was successful in transducing cardiac and skeletal muscle and was able to restore mt‐tRNA^Ala^ levels in skeletal muscle.^142^

The clinical translatability of these approaches remains unclear since cells treated in this way become mtDNA depleted first before endogenous wild‐type mtDNA repopulates. Furthermore, mistargeting of the zinc finger nucleases to the nucleus has been reported.^138^ It also remains to be determined whether shifts in heteroplasmy are sustained over time.

### 6.3 Curing the mitochondrial genome 2: Allotopic expression of mitochondrial proteins

When considering gene therapy for disorders caused by mutations in the 13 protein‐coding mtDNA genes, another challenge is ensuring intra‐mitochondrial expression of the transgene's protein product. One approach, rather than delivering the transgene itself across the mitochondrial membranes, is to express the mitochondrial gene allotopically within the nucleus, and designing the transgene cassette to contain a mitochondrial targeting sequence (MTS) that enables the newly translated polypeptide to enter the mitochondrion through endogenous import mechanisms.^143^ This approach has reached clinical trials in patients with LHON.^144^, ^145^ The most prevalent cause of LHON is a mutation in the *MT‐ND4* gene that encodes a subunit of complex I. Ganglion cells within the retina are most affected in LHON, typically resulting in subacute loss of vision. In one phase I/II study (NCT02064569) which recruited patients in France, rAAV2/2‐*ND4* (including a MTS derived from the COX10 complex IV assembly factor) was administered intravitreally unilaterally to an affected eye in four dose escalations from 9 × 10^9^ to 9 × 10^10^ vg/eye and followed up over a period of almost 5 years.^144^ Adverse events following treatment included anterior chamber inflammation, vitritis and elevated intraocular pressure. In most cases these were managed with appropriate topical anti‐inflammatory agents. Since the gene therapy was delivered unilaterally each patient had a fellow (control) eye for comparison. Best corrected visual acuity improved in the treated eye in 43% of individuals with better outcomes seen in patients with a shorter disease course and a better baseline visual acuity. An alternative strategy being trialled uses a different MTS, derived from the P1 isoform of ATP synthase subunit c, and a recombinant self‐complementary AAV2 scaffold. An open‐label phase I clinical trial (NCT02161380) of the scAAV2‐P1*ND4*v2 vector with three dose escalations is ongoing. Data for the low and medium doses are available.^145^ Low (5 × 10^9^) and medium (2.46 × 10^10^ vg/eye) doses of scAAV2‐P1*ND4*v2 were administered unilaterally to 14 patients with either acute or chronic bilateral visual loss. Comparison was made between mean visual acuity in both injected and fellow eyes compared with baseline. Overall, both injected and fellow eyes demonstrated improvements in visual acuity over follow up, but the improvement seen in injected eyes was greater than that seen in fellow eyes. This effect was more evident in the acute visual loss group than the chronic visual loss group. In those for whom improvement was seen, a rapid treatment effect was noted within 1 month of gene therapy administration, with ongoing improvements in visual acuity over 18 months of follow‐up. The only adverse event noted was anterior uveitis which was mild in all cases and did not require treatment. A high dose escalation to 10^11^ vg/eye is currently recruiting.

## 7 CONCLUSION

This review has discussed pharmacological and genetic therapies for mitochondrial disease, spanning the spectrum from treatments still at a preclinical phase of development to those that have reached phase III clinical trials. Traditionally pharmacological therapies for mitochondrial disease have taken a generic approach, targeting mitochondrial biogenesis, lipid membranes, ROS, and mitophagy. However, for some diseases specific pharmacological approaches are underway, for example nucleoside replacement for TK2 deficiency and enzyme replacement for MNGIE. Genetic therapies are likely to be the most promising approaches ultimately, although most of these are currently at preclinical stages of development. However, as there are at least 350 known mitochondrial diseases, each with its own genetic cause, in future decisions will need to be made as to which are good candidates for gene therapy. Factors to be considered will include disease prevalence (or more precisely the number of patients who could benefit from treatment), the presence of a clinically relevant animal model, and the penetrance of disease as determined by genotype‐phenotype correlation from the natural history.

The significant remaining challenges for trial design should not be underestimated. Several mitochondrial disease trials are actively recruiting but these are mainly early phase (I/II) trials targeting adults with mitochondrial myopathy. There remains a dearth of clinical trials specifically targeting paediatric mitochondrial diseases. Although there are still no curative therapies for the vast majority of individuals affected by primary mitochondrial diseases, it should be remembered that supportive therapies might be lifesaving or life preserving. The last 5 years have seen dramatic changes in the field of mitochondrial medicine, with increased diagnostic power achieved through next generation sequencing approaches. It is hoped that the next 5 years will finally bring licensed disease‐modifying medicines for people affected by mitochondrial disease.

## COMPETING INTERESTS

S.R. is an Investigator on the EE‐TP clinical trial (NCT03866954). R.D.S.P. is Chief Investigator for the clinical trial assessing KL1333 (NeuroVive Pharmaceutical AB, NCT03888716) and Principal Investigator for the REN001‐101 (REN001, Reneo Pharmaceuticals Inc., NCT03862846) and MMPOWER‐3 (Elamipretide, Stealth BioTherapeutics Inc., NCT03323749) clinical trials. R.D.S.P. has received honoraria from Stealth BioTherapeutics and Reneo Pharmaceuticals for S.A.B. meetings. N.K. and J.R. declare no competing interests.

## AUTHOR CONTRIBUTIONS

Drafting manuscript: all authors; Figures: N.K., Table: J.R., S.R.; Review and editing of manuscript: all authors.

## References

[1] Gorman GS, Schaefer AM, Ng Y, et al. Prevalence of nuclear and mitochondrial DNA mutations related to adult mitochondrial disease. Ann Neurol. 2015;77(5):753‐759. 25652200 10.1002/ana.24362 PMC4737121

[2] Rahman J, Rahman S. Mitochondrial medicine in the omics era. Lancet. 2018;391(10139):2560‐2574. 29903433 10.1016/S0140-6736(18)30727-X

[3] Rahman S. Mitochondrial disease in children. J Intern Med. 2020;287:609‐633. 32176382 10.1111/joim.13054

[4] Dogan Sukru A, Pujol C, Maiti P, et al. Tissue‐specific loss of DARS2 activates stress responses independently of respiratory chain deficiency in the heart. Cell Metab. 2014;19(3):458‐469. 24606902 10.1016/j.cmet.2014.02.004

[5] Kauppila JHK, Baines HL, Bratic A, et al. A phenotype‐driven approach to generate mouse models with pathogenic mtDNA mutations causing mitochondrial disease. Cell Rep. 2016;16(11):2980‐2990. 27626666 10.1016/j.celrep.2016.08.037 PMC5039181

[6] Kruse SE, Watt WC, Marcinek DJ, Kapur RP, Schenkman KA, Palmiter RD. Mice with mitochondrial complex I deficiency develop a fatal Encephalomyopathy. Cell Metab. 2008;7(4):312‐320. 18396137 10.1016/j.cmet.2008.02.004 PMC2593686

[7] Nikkanen J, Forsström S, Euro L, et al. Mitochondrial DNA replication defects disturb cellular dNTP pools and remodel one‐carbon metabolism. Cell Metab. 2016;23(4):635‐648. 26924217 10.1016/j.cmet.2016.01.019

[8] Tiranti V, Viscomi C, Hildebrandt T, et al. Loss of ETHE1, a mitochondrial dioxygenase, causes fatal sulfide toxicity in ethylmalonic encephalopathy. Nat Med. 2009;15(2):200‐205. 19136963 10.1038/nm.1907

[9] Garcia‐Corzo L, Luna‐Sanchez M, Doerrier C, et al. Dysfunctional Coq9 protein causes predominant encephalomyopathy associated with CoQ deficiency. Hum Mol Genet. 2013;22(6):1233‐1248. 23255162 10.1093/hmg/dds530

[10] Fassone E, Wedatilake Y, DeVile CJ, Chong WK, Carr LJ, Rahman S. Treatable Leigh‐like encephalopathy presenting in adolescence. BMJ Case Rep. 2013;2013:200838. 10.1136/bcr-2013-200838 PMC3822156 24099834

[11] Balasubramaniam S, Christodoulou J, Rahman S. Disorders of riboflavin metabolism. J Inherit Metab Dis. 2019;42(4):608‐619. 30680745 10.1002/jimd.12058

[12] Olsen RKJ, Konarikova E, Giancaspero TA, et al. Riboflavin‐responsive and ‐non‐responsive mutations in FAD synthase cause multiple acyl‐CoA dehydrogenase and combined respiratory‐chain deficiency. Am J Hum Genet. 2016;98(6):1130‐1145. 27259049 10.1016/j.ajhg.2016.04.006 PMC4908180

[13] O'Callaghan B, Bosch AM, Houlden H. An update on the genetics, clinical presentation, and pathomechanisms of human riboflavin transporter deficiency. J Inherit Metab Dis. 2019;42(4):598‐607. 30793323 10.1002/jimd.12053

[14] Spagnoli C, Pitt MC, Rahman S, de Sousa C. Brown‐Vialetto‐van Laere syndrome: a riboflavin responsive neuronopathy of infancy with singular features. Eur J Paediatr Neurol. 2014;18(2):231‐234. 24206674 10.1016/j.ejpn.2013.09.006

[15] Alcazar‐Fabra M, Trevisson E, Brea‐Calvo G. Clinical syndromes associated with coenzyme Q10 deficiency. Essays Biochem. 2018;62(3):377‐398. 30030365 10.1042/EBC20170107

[16] Montini G, Malaventura C, Salviati L. Early coenzyme Q10 supplementation in primary coenzyme Q10 deficiency. N Engl J Med. 2008;358(26):2849‐2850. 18579827 10.1056/NEJMc0800582

[17] Ashraf S, Gee HY, Woerner S, et al. ADCK4 mutations promote steroid‐resistant nephrotic syndrome through CoQ10 biosynthesis disruption. J Clin Invest. 2013;123(12):5179‐5189. 24270420 10.1172/JCI69000 PMC3859425

[18] Brea‐Calvo G, Haack TB, Karall D, et al. COQ4 mutations cause a broad spectrum of mitochondrial disorders associated with CoQ10 deficiency. Am J Hum Genet. 2015;96(2):309‐317. 25658047 10.1016/j.ajhg.2014.12.023 PMC4320255

[19] Duncan AJ, Bitner‐Glindzicz M, Meunier B, et al. A nonsense mutation in COQ9 causes autosomal‐recessive neonatal‐onset primary coenzyme Q10 deficiency: a potentially treatable form of mitochondrial disease. Am J Hum Genet. 2009;84(5):558‐566. 19375058 10.1016/j.ajhg.2009.03.018 PMC2681001

[20] Awad AM, Bradley MC, Fernandez‐Del‐Rio L, Nag A, Tsui HS, Clarke CF. Coenzyme Q10 deficiencies: pathways in yeast and humans. Essays Biochem. 2018;62(3):361‐376. 29980630 10.1042/EBC20170106 PMC6056717

[21] Pfeffer G, Majamaa K, Turnbull DM, Thorburn D, Chinnery PF. Treatment for mitochondrial disorders. Cochrane Database Syst Rev. 2012;(4):CD004426. 22513923 10.1002/14651858.CD004426.pub3 PMC7201312

[22] Parikh S, Karaa A, Goldstein A, et al. Solid organ transplantation in primary mitochondrial disease: proceed with caution. Mol Genet Metab. 2016;118(3):178‐184. 27312126 10.1016/j.ymgme.2016.04.009

[23] Keshavan N, Rahman S. Natural history of mitochondrial disorders: a systematic review. Essays Biochem. 2018;62(3):423‐442. 29980629 10.1042/EBC20170108

[24] Sofou K, De Coo IF, Isohanni P, et al. A multicenter study on Leigh syndrome: disease course and predictors of survival. Orphanet J Rare Dis. 2014;9:52. 24731534 10.1186/1750-1172-9-52 PMC4021638

[25] Koene S, Rodenburg RJ, van der Knaap MS, et al. Natural disease course and genotype‐phenotype correlations in complex I deficiency caused by nuclear gene defects: what we learned from 130 cases. J Inherit Metab Dis. 2012;35(5):737‐747. 22644603 10.1007/s10545-012-9492-z PMC3432203

[26] Mancuso M, Orsucci D, Angelini C, et al. Redefining phenotypes associated with mitochondrial DNA single deletion. J Neurol. 2015;262(5):1301‐1309. 25808502 10.1007/s00415-015-7710-y

[27] Garone C, Tadesse S, Hirano M. Clinical and genetic spectrum of mitochondrial neurogastrointestinal encephalomyopathy. Brain. 2011;134(11):3326‐3332. 21933806 10.1093/brain/awr245 PMC3212717

[28] Patel KP, O'Brien TW, Subramony SH, Shuster J, Stacpoole PW. The spectrum of pyruvate dehydrogenase complex deficiency: clinical, biochemical and genetic features in 371 patients. Mol Genet Metab. 2012;105(1):34‐43. 22079328 10.1016/j.ymgme.2011.09.032 PMC3754811

[29] Wedatilake Y, Brown RM, McFarland R, et al. SURF1 deficiency: a multi‐centre natural history study. Orphanet J Rare Dis. 2013;8:96. 23829769 10.1186/1750-1172-8-96 PMC3706230

[30] Hikmat O, Naess K, Engvall M, et al. Simplifying the clinical classification of polymerase gamma (POLG) disease based on age of onset; studies using a cohort of 155 cases. J Inherit Metab Dis. 2020;43(4):726–736. 32391929 10.1002/jimd.12211

[31] Grier J, Hirano M, Karaa A, Shepard E, Thompson JLP. Diagnostic odyssey of patients with mitochondrial disease: results of a survey. Neurol Genet. 2018;4(2):e230. 29600276 10.1212/NXG.0000000000000230 PMC5873725

[32] Kaufmann P, Engelstad K, Wei Y, et al. Natural history of MELAS associated with mitochondrial DNA m.3243A>G genotype. Neurology. 2011;77(22):1965‐1971. 22094475 10.1212/WNL.0b013e31823a0c7f PMC3235358

[33] Rajakulendran S, Pitceathly RD, Taanman JW, et al. A clinical, Neuropathological and genetic study of homozygous A467T POLG‐related mitochondrial disease. PLoS ONE. 2016;11(1):e0145500. 26735972 10.1371/journal.pone.0145500 PMC4703200

[34] Grady JP, Pickett SJ, Ng YS, et al. mtDNA heteroplasmy level and copy number indicate disease burden in m.3243A>G mitochondrial disease. EMBO Mol Med. 2018;10(6):e8262. 29735722 10.15252/emmm.201708262 PMC5991564

[35] Nesbitt V, Pitceathly RD, Turnbull DM, et al. The UKMRC mitochondrial disease patient cohort study: clinical phenotypes associated with the m.3243A>G mutation‐‐implications for diagnosis and management. J Neurol Neurosurg Psychiatry. 2013;84(8):936‐938. 23355809 10.1136/jnnp-2012-303528

[36] Mancuso M, Orsucci D, Angelini C, et al. Phenotypic heterogeneity of the 8344A>G mtDNA “MERRF” mutation. Neurology. 2013;80(22):2049‐2054. 23635963 10.1212/WNL.0b013e318294b44c

[37] Barca E, Long Y, Cooley V, et al. Mitochondrial diseases in North America: an analysis of the NAMDC registry. Neurol Genet. 2020;6(2):e402. 32337332 10.1212/NXG.0000000000000402 PMC7164977

[38] Koene S, van Bon L, Bertini E, et al. Outcome measures for children with mitochondrial disease: consensus recommendations for future studies from a Delphi‐based international workshop. J Inherit Metab Dis. 2018;41(6):1267‐1273. 30027425 10.1007/s10545-018-0229-5 PMC6326961

[39] Mancuso M, McFarland R, Klopstock T, Hirano M. Consortium on trial readiness in mitochondrial M. international workshop:: outcome measures and clinical trial readiness in primary mitochondrial myopathies in children and adults. Consensus recommendations. 16‐18 November 2016, Rome, Italy. Neuromuscul Disord. 2017;27(12):1126‐1137. 29074296 10.1016/j.nmd.2017.08.006 PMC6094160

[40] Karaa A, Rahman S, Lombes A, et al. Common data elements for clinical research in mitochondrial disease: a National Institute for neurological disorders and stroke project. J Inherit Metab Dis. 2017;40(3):403‐414. 28303425 10.1007/s10545-017-0035-5 PMC7783474

[41] Lehtonen JM, Forsstrom S, Bottani E, et al. FGF21 is a biomarker for mitochondrial translation and mtDNA maintenance disorders. Neurology. 2016;87(22):2290‐2299. 27794108 10.1212/WNL.0000000000003374 PMC5270510

[42] Boenzi S, Diodato D. Biomarkers for mitochondrial energy metabolism diseases. Essays Biochem. 2018;62(3):443‐454. 29980631 10.1042/EBC20170111

[43] Pfeffer G, Horvath R, Klopstock T, et al. New treatments for mitochondrial disease‐no time to drop our standards. Nat Rev Neurol. 2013;9(8):474‐481. 23817350 10.1038/nrneurol.2013.129 PMC4967498

[44] Zolkipli‐Cunningham Z, Xiao R, Stoddart A, et al. Mitochondrial disease patient motivations and barriers to participate in clinical trials. PLoS ONE. 2018;13(5):e0197513. 29771953 10.1371/journal.pone.0197513 PMC5957366

[45] Guicciardi ME, Gores GJ. Apoptosis: a mechanism of acute and chronic liver injury. Gut. 2005;54(7):1024‐1033. 15951554 10.1136/gut.2004.053850 PMC1774601

[46] Kinnunen PKJ, Kaarniranta K, Mahalka AK. Protein‐oxidized phospholipid interactions in cellular signaling for cell death: from biophysics to clinical correlations. Biochim Biophys Acta Biomembr. 2012;1818(10):2446‐2455. 10.1016/j.bbamem.2012.04.008 22542574

[47] Dogan SA, Cerutti R, Beninca C, et al. Perturbed redox signaling exacerbates a mitochondrial myopathy. Cell Metab. 2018;28(5):764‐775 e765. 30122554 10.1016/j.cmet.2018.07.012 PMC6224544

[48] Jaber S, Polster BM. Idebenone and neuroprotection: antioxidant, pro‐oxidant, or electron carrier? J Bioenerg Biomembr. 2015;47(1–2):111‐118. 25262284 10.1007/s10863-014-9571-y PMC4487815

[49] Klopstock T, Yu‐Wai‐Man P, Dimitriadis K, et al. A randomized placebo‐controlled trial of idebenone in Leber's hereditary optic neuropathy. Brain. 2011;134(9):2677‐2686. 21788663 10.1093/brain/awr170 PMC3170530

[50] Martinelli D, Catteruccia M, Piemonte F, et al. EPI‐743 reverses the progression of the pediatric mitochondrial disease–genetically defined Leigh syndrome. Mol Genet Metab. 2012;107(3):383‐388. 23010433 10.1016/j.ymgme.2012.09.007

[51] Enns GM, Kinsman SL, Perlman SL, et al. Initial experience in the treatment of inherited mitochondrial disease with EPI‐743. Mol Genet Metab. 2012;105(1):91‐102. 22115768 10.1016/j.ymgme.2011.10.009

[52] Beyrath J, Pellegrini M, Renkema H, et al. KH176 safeguards mitochondrial diseased cells from redox stress‐induced cell death by interacting with the Thioredoxin system/Peroxiredoxin enzyme machinery. Sci Rep. 2018;8(1):6577. 29700325 10.1038/s41598-018-24900-3 PMC5920042

[53] de Haas R, Das D, Garanto A, et al. Therapeutic effects of the mitochondrial ROS‐redox modulator KH176 in a mammalian model of Leigh disease. Sci Rep. 2017;7(1):11733. 28916769 10.1038/s41598-017-09417-5 PMC5601915

[54] Janssen MCH, Koene S, de Laat P, et al. The KHENERGY study: safety and efficacy of KH176 in mitochondrial m.3243A>G Spectrum disorders. Clin Pharmacol Ther. 2019;105(1):101‐111. 30058726 10.1002/cpt.1197 PMC6704357

[55] Spiegelman BM. Transcriptional control of mitochondrial energy metabolism through the PGC1 coactivators. Novartis Found Symp. 2007;287:60‐63. discussion 63‐69. 18074631

[56] Komen JC, Thorburn DR. Turn up the power ‐ pharmacological activation of mitochondrial biogenesis in mouse models. Br J Pharmacol. 2014;171(8):1818‐1836. 24102298 10.1111/bph.12413 PMC3976607

[57] Bogacka I, Xie H, Bray GA, Smith SR. Pioglitazone induces mitochondrial biogenesis in human subcutaneous adipose tissue in vivo. Diabetes. 2005;54(5):1392‐1399. 15855325 10.2337/diabetes.54.5.1392

[58] Lagouge M, Argmann C, Gerhart‐Hines Z, et al. Resveratrol improves mitochondrial function and protects against metabolic disease by activating SIRT1 and PGC‐1alpha. Cell. 2006;127(6):1109‐1122. 17112576 10.1016/j.cell.2006.11.013

[59] Reisman SA, Gahir SS, Lee CI, Proksch JW, Sakamoto M, Ward KW. Pharmacokinetics and pharmacodynamics of the novel Nrf2 activator omaveloxolone in primates. Drug Des Devel Ther. 2019;13:1259‐1270. 10.2147/DDDT.S193889 PMC6475100 31118567

[60] Viscomi C, Bottani E, Civiletto G, et al. In vivo correction of COX deficiency by activation of the AMPK/PGC‐1alpha axis. Cell Metab. 2011;14(1):80‐90. 21723506 10.1016/j.cmet.2011.04.011 PMC3130927

[61] Cerutti R, Pirinen E, Lamperti C, et al. NAD(+)‐dependent activation of Sirt1 corrects the phenotype in a mouse model of mitochondrial disease. Cell Metab. 2014;19(6):1042‐1049. 24814483 10.1016/j.cmet.2014.04.001 PMC4051987

[62] Khan NA, Auranen M, Paetau I, et al. Effective treatment of mitochondrial myopathy by nicotinamide riboside, a vitamin B3. EMBO Mol Med. 2014;6(6):721‐731. 24711540 10.1002/emmm.201403943 PMC4203351

[63] Kanabus M, Fassone E, Hughes SD, et al. The pleiotropic effects of decanoic acid treatment on mitochondrial function in fibroblasts from patients with complex I deficient Leigh syndrome. J Inherit Metab Dis. 2016;39(3):415‐426. 27080638 10.1007/s10545-016-9930-4 PMC4851692

[64] Gueguen N, Desquiret‐Dumas V, Leman G, et al. Resveratrol directly binds to mitochondrial complex I and increases oxidative stress in brain mitochondria of aged mice. PLoS ONE. 2015;10(12):e0144290. 26684010 10.1371/journal.pone.0144290 PMC4694087

[65] Yatsuga S, Suomalainen A. Effect of bezafibrate treatment on late‐onset mitochondrial myopathy in mice. Hum Mol Genet. 2012;21(3):526‐535. 22012983 10.1093/hmg/ddr482

[66] Steele H, Gomez‐Duran A, Pyle A, et al. Metabolic effects of bezafibrate in mitochondrial disease. EMBO Mol Med. 2020;12(3):e11589. 32107855 10.15252/emmm.201911589 PMC7059007

[67] Madsen KL, Buch AE, Cohen BH, et al. Safety and efficacy of omaveloxolone in patients with mitochondrial myopathy: MOTOR trial. Neurology. 2020;94(7):e687‐e698. 31896620 10.1212/WNL.0000000000008861 PMC7176297

[68] Pirinen E, Auranen M, Khan NA, et al. Niacin cures systemic NAD(+) deficiency and improves muscle performance in adult‐onset mitochondrial myopathy. Cell Metab. 2020;31:1078‐1090.e5. 32386566 10.1016/j.cmet.2020.04.008

[69] Karaa A, Haas R, Goldstein A, Vockley J, Cohen BH. A randomized crossover trial of elamipretide in adults with primary mitochondrial myopathy. J Cachexia Sarcopenia Muscle. 2020. [online ahead of print] 10.1002/jcsm.12559 PMC7432581 32096613

[70] Stealth BioTherapeutics Inc. provides update on Phase III Trial of Elamipretide in Primary Mitochondrial Myopathy. 2019. https://www.prnewswire.com/news-releases/stealth-biotherapeutics-provides-update-on-phase-3-trial-of-elamipretide-in-primary-mitochondrial-myopathy-300978082.html.

[71] Thompson RMR, Aiudi A, Jones JJ, Carr J, Hornby B, Vernon H. Elamipretide in patients with Barth syndrome: a randomized, double‐blind, placebo‐controlled clinical trial followed by 36‐week open‐label extension. J Am Coll Cardiol. 2020;75(11) Supp 1:957.

[72] Ashrafi G, Schwarz TL. The pathways of mitophagy for quality control and clearance of mitochondria. Cell Death Differ. 2013;20(1):31‐42. 22743996 10.1038/cdd.2012.81 PMC3524633

[73] Gilkerson RW, De Vries RL, Lebot P, et al. Mitochondrial autophagy in cells with mtDNA mutations results from synergistic loss of transmembrane potential and mTORC1 inhibition. Hum Mol Genet. 2012;21(5):978‐990. 22080835 10.1093/hmg/ddr529 PMC3277306

[74] Johnson SC, Yanos ME, Kayser EB, et al. mTOR inhibition alleviates mitochondrial disease in a mouse model of Leigh syndrome. Science. 2013;342(6165):1524‐1528. 24231806 10.1126/science.1244360 PMC4055856

[75] Civiletto G, Dogan SA, Cerutti R, et al. Rapamycin rescues mitochondrial myopathy via coordinated activation of autophagy and lysosomal biogenesis. EMBO Mol Med. 2018;10(11):e8799. 30309855 10.15252/emmm.201708799 PMC6220341

[76] Siegmund SE, Yang H, Sharma R, et al. Low‐dose rapamycin extends lifespan in a mouse model of mtDNA depletion syndrome. Hum Mol Genet. 2017;26(23):4588‐4605. 28973153 10.1093/hmg/ddx341 PMC5886265

[77] Khan NA, Nikkanen J, Yatsuga S, et al. mTORC1 regulates mitochondrial integrated stress response and mitochondrial myopathy progression. Cell Metab. 2017;26(2):419‐428 e415. 28768179 10.1016/j.cmet.2017.07.007

[78] Wang A, Mouser J, Pitt J, Promislow D, Kaeberlein M. Rapamycin enhances survival in a drosophila model of mitochondrial disease. Oncotarget. 2016;7(49):80131‐80139. 27741510 10.18632/oncotarget.12560 PMC5348310

[79] Zheng X, Boyer L, Jin M, et al. Alleviation of neuronal energy deficiency by mTOR inhibition as a treatment for mitochondria‐related neurodegeneration. elife. 2016;5:e13378. 27008180 10.7554/eLife.13378 PMC4846388

[80] Peng M, Ostrovsky J, Kwon YJ, et al. Inhibiting cytosolic translation and autophagy improves health in mitochondrial disease. Hum Mol Genet. 2015;24(17):4829‐4847. 26041819 10.1093/hmg/ddv207 PMC4527487

[81] Barriocanal‐Casado E, Hidalgo‐Gutierrez A, Raimundo N, et al. Rapamycin administration is not a valid therapeutic strategy for every case of mitochondrial disease. EBioMedicine. 2019;42:511‐523. 30898651 10.1016/j.ebiom.2019.03.025 PMC6492073

[82] Johnson SC, Martinez F, Bitto A, et al. mTOR inhibitors may benefit kidney transplant recipients with mitochondrial diseases. Kidney Int. 2019;95(2):455‐466. 30471880 10.1016/j.kint.2018.08.038 PMC6389356

[83] Sage‐Schwaede A, Engelstad K, Salazar R, et al. Exploring mTOR inhibition as treatment for mitochondrial disease. Ann Clin Transl Neurol. 2019;6(9):1877‐1881. 31386302 10.1002/acn3.50846 PMC6764630

[84] Sanz A, Soikkeli M, Portero‐Otin M, et al. Expression of the yeast NADH dehydrogenase Ndi1 in drosophila confers increased lifespan independently of dietary restriction. Proc Natl Acad Sci U S A. 2010;107(20):9105‐9110. 20435911 10.1073/pnas.0911539107 PMC2889079

[85] Dassa EP, Dufour E, Goncalves S, et al. Expression of the alternative oxidase complements cytochrome c oxidase deficiency in human cells. EMBO Mol Med. 2009;1(1):30‐36. 20049701 10.1002/emmm.200900001 PMC3378104

[86] Fernandez‐Ayala DJ, Sanz A, Vartiainen S, et al. Expression of the *Ciona intestinalis* alternative oxidase (AOX) in drosophila complements defects in mitochondrial oxidative phosphorylation. Cell Metab. 2009;9(5):449‐460. 19416715 10.1016/j.cmet.2009.03.004

[87] Perales‐Clemente E, Bayona‐Bafaluy MP, Perez‐Martos A, Barrientos A, Fernandez‐Silva P, Enriquez JA. Restoration of electron transport without proton pumping in mammalian mitochondria. Proc Natl Acad Sci U S A. 2008;105(48):18735‐18739. 19020091 10.1073/pnas.0810518105 PMC2585044

[88] Rajendran J, Purhonen J, Tegelberg S, et al. Alternative oxidase‐mediated respiration prevents lethal mitochondrial cardiomyopathy. EMBO Mol Med. 2019;11(1):e9456. 30530468 10.15252/emmm.201809456 PMC6328925

[89] Patgiri A, Skinner OS, Miyazaki Y, et al. An engineered enzyme that targets circulating lactate to alleviate intracellular NADH:NAD(+) imbalance. Nat Biotechnol. 2020;38(3):309‐313. 31932725 10.1038/s41587-019-0377-7 PMC7135927

[90] Jain IH, Zazzeron L, Goli R, et al. Hypoxia as a therapy for mitochondrial disease. Science. 2016;352(6281):54‐61. 26917594 10.1126/science.aad9642 PMC4860742

[91] Mootha VK, Chinnery PF. Oxygen in mitochondrial disease: can there be too much of a good thing? J Inherit Metab Dis. 2018;41(5):761‐763. 29948481 10.1007/s10545-018-0210-3

[92] Peters MJ, Jones GA, Eaton S, Wiley D, Ray S. Risks and benefits of oxygen therapy. J Inherit Metab Dis. 2018;41(5):757‐759. 29869161 10.1007/s10545-018-0208-x

[93] Lopez‐Gomez C, Levy RJ, Sanchez‐Quintero MJ, et al. Deoxycytidine and Deoxythymidine treatment for thymidine kinase 2 deficiency. Ann Neurol. 2017;81(5):641‐652. 28318037 10.1002/ana.24922 PMC5926768

[94] Garone C, Taylor RW, Nascimento A, et al. Retrospective natural history of thymidine kinase 2 deficiency. J Med Genet. 2018;55(8):515‐521. 29602790 10.1136/jmedgenet-2017-105012 PMC6073909

[95] Brinkman K, ter Hofstede HJM, Burger DM, Smeitink JAM, Koopmans PP. Adverse effects of reverse transcriptase inhibitors: mitochondrial toxicity as common pathway. AIDS. 1998;12(14):1735‐1744. 9792373 10.1097/00002030-199814000-00004

[96] Johnson AA, Ray AS, Hanes J, et al. Toxicity of antiviral nucleoside analogs and the human mitochondrial DNA polymerase. J Biol Chem. 2001;276(44):40847‐40857. 11526116 10.1074/jbc.M106743200

[97] Copeland WC. The mitochondrial DNA polymerase in health and disease. Subcell Biochem. 2010;50:211‐222. 20012584 10.1007/978-90-481-3471-7_11 PMC3960799

[98] Arnaudo E, Dalakas M, Shanske S, Moraes CT, DiMauro S, Schon EA. Depletion of muscle mitochondrial DNA in AIDS patients with zidovudine‐induced myopathy. Lancet. 1991;337(8740):508‐510. 1671889 10.1016/0140-6736(91)91294-5

[99] Halter JP, Michael W, Schupbach M, et al. Allogeneic haematopoietic stem cell transplantation for mitochondrial neurogastrointestinal encephalomyopathy. Brain. 2015;138(10):2847‐2858. 26264513 10.1093/brain/awv226 PMC4836400

[100] Bax BE, Levene M, Bain MD, et al. Erythrocyte encapsulated thymidine Phosphorylase for the treatment of patients with mitochondrial Neurogastrointestinal Encephalomyopathy: study protocol for a multi‐Centre, multiple dose, open label trial. J Clin Med. 2019;8(8):1096. 10.3390/jcm8081096 PMC6722784 31344955

[101] Boschetti E, D'Alessandro R, Bianco F, et al. Liver as a source for thymidine phosphorylase replacement in mitochondrial neurogastrointestinal encephalomyopathy. PLoS ONE. 2014;9(5):e96692. 24802030 10.1371/journal.pone.0096692 PMC4011889

[102] Grabhorn E, Tsiakas K, Herden U, et al. Long‐term outcomes after liver transplantation for deoxyguanosine kinase deficiency: a single‐center experience and a review of the literature. Liver Transpl. 2014;20(4):464‐472. 24478274 10.1002/lt.23830

[103] Diomedi‐Camassei F, Di Giandomenico S, Santorelli FM, et al. COQ2 nephropathy: a newly described inherited mitochondriopathy with primary renal involvement. J Am Soc Nephrol. 2007;18(10):2773‐2780. 17855635 10.1681/ASN.2006080833

[104] Homan DJ, Niyazov DM, Fisher PW, et al. Heart transplantation for a patient with Kearns‐Sayre syndrome and end‐stage heart failure. Congest Heart Fail. 2011;17(2):102‐104. 21450000 10.1111/j.1751-7133.2011.00211.x

[105] Bonnefoy N, Fox TD. Directed alteration of *Saccharomyces cerevisiae* mitochondrial DNA by biolistic transformation and homologous recombination. Methods Mol Biol. 2007;372:153‐166. 18314724 10.1007/978-1-59745-365-3_11 PMC2771616

[106] Yasuzaki Y, Yamada Y, Ishikawa T, Harashima H. Validation of mitochondrial gene delivery in liver and skeletal muscle via hydrodynamic injection using an artificial mitochondrial reporter DNA vector. Mol Pharm. 2015;12(12):4311‐4320. 26567847 10.1021/acs.molpharmaceut.5b00511

[107] Yamada Y, Furukawa R, Yasuzaki Y, Harashima H. Dual function MITO‐porter, a nano carrier integrating both efficient cytoplasmic delivery and mitochondrial macromolecule delivery. Mol Ther. 2011;19(8):1449‐1456. 21694702 10.1038/mt.2011.99 PMC3149179

[108] Santos J, Sousa F, Queiroz J, Costa D. Rhodamine based plasmid DNA nanoparticles for mitochondrial gene therapy. Colloids Surf B Biointerfaces. 2014;121:129‐140. 24967548 10.1016/j.colsurfb.2014.06.003

[109] Flierl A, Jackson C, Cottrell B, Murdock D, Seibel P, Wallace DC. Targeted delivery of DNA to the mitochondrial compartment via import sequence‐conjugated peptide nucleic acid. Mol Ther. 2003;7(4):550‐557. 12727119 10.1016/s1525-0016(03)00037-6

[110] Jang YH, Lim KI. Recent advances in mitochondria‐targeted gene delivery. Molecules. 2018;23(9):2316. 10.3390/molecules23092316 PMC6225103 30208599

[111] Zincarelli C, Soltys S, Rengo G, Rabinowitz JE. Analysis of AAV serotypes 1‐9 mediated gene expression and tropism in mice after systemic injection. Mol Ther. 2008;16(6):1073‐1080. 18414476 10.1038/mt.2008.76

[112] Zhang H, Yang B, Mu X, et al. Several rAAV vectors efficiently cross the blood‐brain barrier and transduce neurons and astrocytes in the neonatal mouse central nervous system. Mol Ther. 2011;19(8):1440‐1448. 21610699 10.1038/mt.2011.98 PMC3149178

[113] Schnepp BC, Clark KR, Klemanski DL, Pacak CA, Johnson PR. Genetic fate of recombinant adeno‐associated virus vector genomes in muscle. J Virol. 2003;77(6):3495‐3504. 12610125 10.1128/JVI.77.6.3495-3504.2003 PMC149530

[114] Zaiss AK, Liu Q, Bowen GP, Wong NC, Bartlett JS, Muruve DA. Differential activation of innate immune responses by adenovirus and adeno‐associated virus vectors. J Virol. 2002;76(9):4580‐4590. 11932423 10.1128/JVI.76.9.4580-4590.2002 PMC155101

[115] Murrey DA, Naughton BJ, Duncan FJ, et al. Feasibility and safety of systemic rAAV9‐hNAGLU delivery for treating mucopolysaccharidosis IIIB: toxicology, biodistribution, and immunological assessments in primates. Hum Gene Ther Clin Dev. 2014;25(2):72‐84. 24720466 10.1089/humc.2013.208 PMC4124586

[116] Benkhelifa‐Ziyyat S, Besse A, Roda M, et al. Intramuscular scAAV9‐SMN injection mediates widespread gene delivery to the spinal cord and decreases disease severity in SMA mice. Mol Ther. 2013;21(2):282‐290. 23295949 10.1038/mt.2012.261 PMC3594018

[117] Doerfler PA, Todd AG, Clement N, et al. Copackaged AAV9 vectors promote simultaneous immune tolerance and phenotypic correction of Pompe disease. Hum Gene Ther. 2016;27(1):43‐59. 26603344 10.1089/hum.2015.103 PMC4741206

[118] Du S, Ou H, Cui R, et al. Delivery of Glucosylceramidase Beta gene using AAV9 vector therapy as a treatment strategy in mouse models of Gaucher disease. Hum Gene Ther. 2019;30(2):155‐167. 30122074 10.1089/hum.2018.072

[119] Laoharawee K, Podetz‐Pedersen KM, Nguyen TT, et al. Prevention of neurocognitive deficiency in Mucopolysaccharidosis type II mice by central nervous system‐directed, AAV9‐mediated Iduronate Sulfatase gene transfer. Hum Gene Ther. 2017;28(8):626‐638. 28478695 10.1089/hum.2016.184

[120] Ribera A, Haurigot V, Garcia M, et al. Biochemical, histological and functional correction of mucopolysaccharidosis type IIIB by intra‐cerebrospinal fluid gene therapy. Hum Mol Genet. 2015;24(7):2078‐2095. 25524704 10.1093/hmg/ddu727

[121] Weismann CM, Ferreira J, Keeler AM, et al. Systemic AAV9 gene transfer in adult GM1 gangliosidosis mice reduces lysosomal storage in CNS and extends lifespan. Hum Mol Genet. 2015;24(15):4353‐4364. 25964428 10.1093/hmg/ddv168 PMC4492398

[122] Mendell JR, Al‐Zaidy S, Shell R, et al. Single‐dose gene‐replacement therapy for spinal muscular atrophy. N Engl J Med. 2017;377(18):1713‐1722. 29091557 10.1056/NEJMoa1706198

[123] Russell S, Bennett J, Wellman JA, et al. Efficacy and safety of voretigene neparvovec (AAV2‐hRPE65v2) in patients with RPE65‐mediated inherited retinal dystrophy: a randomised, controlled, open‐label, phase 3 trial. Lancet. 2017;390(10097):849‐860. 28712537 10.1016/S0140-6736(17)31868-8 PMC5726391

[124] Bottani E, Giordano C, Civiletto G, et al. AAV‐mediated liver‐specific MPV17 expression restores mtDNA levels and prevents diet‐induced liver failure. Mol Ther. 2014;22(1):10‐17. 24247928 10.1038/mt.2013.230 PMC3880585

[125] Di Meo I, Auricchio A, Lamperti C, Burlina A, Viscomi C, Zeviani M. Effective AAV‐mediated gene therapy in a mouse model of ethylmalonic encephalopathy. EMBO Mol Med. 2012;4(9):1008‐1014. 22903887 10.1002/emmm.201201433 PMC3491831

[126] Di Meo I, Marchet S, Lamperti C, Zeviani M, Viscomi C. AAV9‐based gene therapy partially ameliorates the clinical phenotype of a mouse model of Leigh syndrome. Gene Ther. 2017;24(10):661‐667. 28753212 10.1038/gt.2017.53 PMC5658670

[127] Suzuki‐Hatano S, Sriramvenugopal M, Ramanathan M, et al. Increased mtDNA abundance and improved function in human Barth syndrome patient fibroblasts following AAV‐TAZ gene delivery. Int J Mol Sci. 2019;20(14):3416. 10.3390/ijms20143416 PMC6678701 31336787

[128] Yang L, Slone J, Li Z, et al. Systemic administration of AAV‐Slc25a46 mitigates mitochondrial neuropathy in Slc25a46−/− mice. Hum Mol Genet. 2020;29:649‐661. 31943007 10.1093/hmg/ddz277 PMC7068115

[129] Torres‐Torronteras J, Gomez A, Eixarch H, et al. Hematopoietic gene therapy restores thymidine phosphorylase activity in a cell culture and a murine model of MNGIE. Gene Ther. 2011;18(8):795‐806. 21451581 10.1038/gt.2011.24 PMC7568345

[130] Torres‐Torronteras J, Cabrera‐Perez R, Vila‐Julia F, et al. Long‐term sustained effect of liver‐targeted adeno‐associated virus gene therapy for mitochondrial neurogastrointestinal encephalomyopathy. Hum Gene Ther. 2018;29(6):708‐718. 29284302 10.1089/hum.2017.133 PMC7647931

[131] Reynaud‐Dulaurier R, Benegiamo G, Marrocco E, et al. Gene replacement therapy provides benefit in an adult mouse model of Leigh syndrome. Brain. 2020;143:1686‐1696. 32413099 10.1093/brain/awaa105

[132] Liguore WA, Domire JS, Button D, et al. AAV‐PHP.B administration results in a differential pattern of CNS biodistribution in non‐human primates compared with mice. Mol Ther. 2019;27(11):2018‐2037. 31420242 10.1016/j.ymthe.2019.07.017 PMC6838922

[133] Pereira CV, Peralta S, Arguello T, Bacman SR, Diaz F, Moraes CT. Myopathy reversion in mice after restauration of mitochondrial complex I. EMBO Mol Med. 2020;12(2):e10674. 31916679 10.15252/emmm.201910674 PMC7005622

[134] Wang L, Calcedo R, Bell P, et al. Impact of pre‐existing immunity on gene transfer to nonhuman primate liver with adeno‐associated virus 8 vectors. Hum Gene Ther. 2011;22(11):1389‐1401. 21476868 10.1089/hum.2011.031 PMC3225046

[135] Dong JY, Fan PD, Frizzell RA. Quantitative analysis of the packaging capacity of recombinant adeno‐associated virus. Hum Gene Ther. 1996;7(17):2101‐2112. 8934224 10.1089/hum.1996.7.17-2101

[136] Hanna E, Remuzat C, Auquier P, Toumi M. Gene therapies development: slow progress and promising prospect. J Mark Access Health Policy. 2017;5(1):1265293. 28265348 10.1080/20016689.2017.1265293 PMC5328344

[137] Nissanka N, Moraes CT. Mitochondrial DNA heteroplasmy in disease and targeted nuclease‐based therapeutic approaches. EMBO Rep. 2020;21(3):e49612. 32073748 10.15252/embr.201949612 PMC7054667

[138] Minczuk M, Papworth MA, Kolasinska P, Murphy MP, Klug A. Sequence‐specific modification of mitochondrial DNA using a chimeric zinc finger methylase. Proc Natl Acad Sci U S A. 2006;103(52):19689‐19694. 17170133 10.1073/pnas.0609502103 PMC1750892

[139] Gammage PA, Rorbach J, Vincent AI, Rebar EJ, Minczuk M. Mitochondrially targeted ZFNs for selective degradation of pathogenic mitochondrial genomes bearing large‐scale deletions or point mutations. EMBO Mol Med. 2014;6(4):458‐466. 24567072 10.1002/emmm.201303672 PMC3992073

[140] Gammage PA, Viscomi C, Simard ML, et al. Genome editing in mitochondria corrects a pathogenic mtDNA mutation in vivo. Nat Med. 2018;24(11):1691‐1695. 30250142 10.1038/s41591-018-0165-9 PMC6225988

[141] Bacman SR, Williams SL, Pinto M, Peralta S, Moraes CT. Specific elimination of mutant mitochondrial genomes in patient‐derived cells by mitoTALENs. Nat Med. 2013;19(9):1111‐1113. 23913125 10.1038/nm.3261 PMC4153471

[142] Bacman SR, Kauppila JHK, Pereira CV, et al. MitoTALEN reduces mutant mtDNA load and restores tRNA(Ala) levels in a mouse model of heteroplasmic mtDNA mutation. Nat Med. 2018;24(11):1696‐1700. 30250143 10.1038/s41591-018-0166-8 PMC6942693

[143] Gearing DP, Nagley P. Yeast mitochondrial ATPase subunit 8, normally a mitochondrial gene product, expressed in vitro and imported back into the organelle. EMBO J. 1986;5(13):3651‐3655. 2881782 10.1002/j.1460-2075.1986.tb04695.x PMC1167406

[144] Vignal C, Uretsky S, Fitoussi S, et al. Safety of rAAV2/2‐ND4 gene therapy for Leber hereditary optic neuropathy. Ophthalmology. 2018;125(6):945‐947. 29426586 10.1016/j.ophtha.2017.12.036

[145] Guy J, Feuer WJ, Davis JL, et al. Gene therapy for Leber hereditary optic neuropathy: Low‐ and medium‐dose visual results. Ophthalmology. 2017;124(11):1621‐1634. 28647203 10.1016/j.ophtha.2017.05.016 PMC5831379

